# Genetic deletion of G protein-coupled receptor 56 aggravates traumatic brain injury through the microglial CCL3/4/5 upregulation targeted to CCR5

**DOI:** 10.1038/s41419-025-07501-7

**Published:** 2025-03-15

**Authors:** Zhuang Sha, Shiying Dong, Meng Nie, Tao Liu, Chenrui Wu, Chuanxiang Lv, Mingqi Liu, Weiwei Jiang, Jiangyuan Yuan, Yu Qian, Xianhua Piao, Rongcai Jiang, Chuang Gao

**Affiliations:** 1 Department of Neurosurgery, The Affiliated Hospital of Xuzhou Medical University, Xuzhou, China; 2 Department of Neurosurgery, Tianjin Medical University General Hospital, Tianjin, China; 3 Tianjin Neurological Institute, Key Laboratory of Post Neuro-injury Neuro-repair and Regeneration in Central Nervous System, Ministry of Education, Tianjin, China; 4 Weill Institute for Neuroscience, University of California, San Francisco (UCSF), San Francisco, CA USA; 5 Newborn Brain Research Institute, University of California, San Francisco (UCSF), San Francisco, CA USA; 6 Eli and Edythe Broad Center of Regeneration Medicine and Stem Cell Research, University of California, San Francisco (UCSF), San Francisco, CA USA; 7 Division of Neonatology, Department of Pediatrics, University of California, San Francisco (UCSF), San Francisco, CA USA; 8 Department of Neurosurgery, Xuanwu Hospital, Capital Medical University, Beijing, China; 9 State Key Laboratory of Experimental Hematology, Laboratory of Post-Neuroinjury Neurorepair and Regeneration in Central Nervous System Tianjin & Ministry of Education, Tianjin Neurological Institute, Tianjin Medical University General Hospital, Tianjin, China

**Keywords:** Neuroimmunology, Trauma

## Abstract

Traumatic brain injury (TBI) is a significant global health concern that often results in death or disability, and effective pharmacological treatments are lacking. G protein-coupled receptor 56 (GPR56), a potential drug target, is crucial for neuronal and glial cell function and therefore plays important roles in various neurological diseases. Here, we investigated the potential role and mechanism of GPR56 in TBI-related damage to gain new insights into the pharmacological treatment of TBI. Our study revealed that TBI caused a significant decrease in GPR56 expression and that the deletion of *Gpr56* exacerbated neurological function deficits and blood‒brain barrier (BBB) damage following TBI. Additionally, *Gpr56* deletion led to increased microgliosis, increased infiltration of peripheral T cells and macrophages, and increased release of cerebral inflammatory cytokines and chemokines after TBI. Furthermore, *Gpr56* deletion induced neuronal apoptosis, impaired autophagy, and exacerbated neurological function deficits through microglial-to-neuronal CCR5 signaling after TBI. Overall, these results indicate that *Gpr56* knockout exacerbates neurological deficits, neuroinflammation and neuronal apoptosis following TBI through microglial CCL3/4/5 upregulation targeted to CCR5, which indicates that GRP56 may be a potential new pharmacological target for TBI.

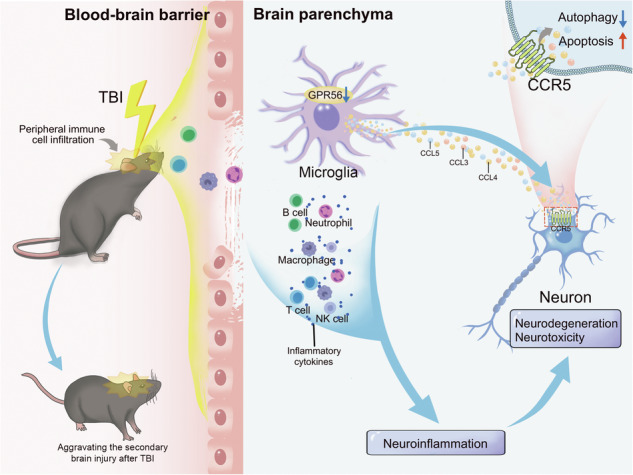

## Introduction

Traumatic brain injury (TBI), arising from various incidents such as traffic accidents, industrial mishaps, recreational activities, falls, and military conflicts, represents a significant and increasing contributor to global disability and mortality [1]. TBI survivors endure neurofunctional deficits, including headaches, slurred speech, aphasia, sensory and motor impairments, cognitive challenges (impacting learning and memory), and emotional dysfunction such as irritability and aggression [2]. Classical pathophysiological features of TBI comprise synaptic dysfunction, neuronal cell death, axonal damage, activation of glial cells, neuroinflammation, and infiltration of immune cells [3–6]. Activation of microglia, astrocytes, and infiltrated immune cells induces the release of numerous inflammatory cytokines and chemokines following trauma [7, 8]. Together, these mechanisms broaden both the extent of neural damage and the range of impairments beyond those caused by the initial insult, referred to as secondary injury. Despite the continuous development of new diagnostic and therapeutic techniques by neurosurgeons and scientists, including hormone pulse therapy, intracranial pressure monitoring, decompressive craniectomy, mild hypothermia treatment, progesterone therapy, etc., few randomized controlled clinical trials (RCTs) have confirmed positive therapeutic results [9]. Therefore, it is crucial to investigate the fundamental molecular mechanisms that exacerbate TBI and identify new potential targets for therapy.

The family of G protein-coupled receptors (GPCRs) represents effective targets for drug discovery, with approximately 1/3 of drugs reported to act on GPCRs [10]. GPR56 is a member of the adhesion G protein-coupled receptor family, the second largest family of GPCRs, which is lack of specific agonists or antagonists currently. The GPR56 gene is located on human chromosome 16q13 and mouse chromosome 8 [11, 12], playing important functions in cell-to-cell and cell–matrix communications during brain development and disorders [13]. During embryonic brain development, GPR56 is expressed in neural progenitor cells and migrating neurons, interacting with its extracellular matrix (ECM) ligand collagen III to orchestrate cortical lamination [12, 14, 15]. As brain development progresses and throughout postnatal life, GPR56 exhibits high expression levels in various glial cell types, including astrocytes, cells of the oligodendrocyte lineage, and microglia [16, 17]. GPR56 is also widely present in peripheral blood on the surface of cytotoxic NK cells and T lymphocytes, regulating peripheral inflammatory responses [18]. NK cell activation leads to a decrease in surface GPR56 expression, and overexpression of GPR56 reduces NK cell cytotoxicity and cytokine production [19], suggesting that GPR56 may inhibit excessive inflammatory responses in immune cells. Microglia are resident macrophages in the central nervous system (CNS), originating from hematopoietic stem cells in the yolk sac. In contrast, macrophages infiltrating brain tissue after TBI originate from the bone marrow. Both types of cells share similar surface markers. Importantly, GPR56 defines yolk sac-derived microglia [20] and is not expressed in peripheral macrophages [21]. In addition, GPR56 plays a promoting role in myelin regeneration, microglial synaptic refinement and development of cerebral cortex [22–27].

In this study, we sought to uncover the critical role of GPR56 in modulating the function of microglial response to TBI and long-term neurological function deficits following TBI.

## Materials and methods

### Animals, drug administration and experimental groups

All experiments were approved by the Animal Care and Use Committee of Tianjin Medical University General Hospital (No. IRB2020-DWFL-028) and were conducted following the Reporting of In Vivo Experiments (ARRIVE) guidelines. Throughout the study period, all mice were housed in the specific pathogen-free (SPF) environment of our laboratory animal center. The animals had ad libitum access to food and water and were maintained under controlled conditions, including an ambient temperature of 22 ± 1°C, relative humidity of 50–60%, and a 12-hour light/dark cycle with lights on at 8:00 a.m. daily. Every possible measure was taken to reduce animal suffering and minimize the necessity for euthanasia. *Gpr56* knockout (KO) mice were kindly provided by Prof. Xianhua Piao [28]. The mutant mice were originally created in a 129/BL6 background, but were rederived into the FvB strain and bred to BALB/c at one point. Therefore, the genetic background of the mutant mice is mixed: 129/BL6/FvB/BALB/c. Genotyping for the GPR56 allele was conducted using polymerase chain reaction (PCR) with the following primers: Primer 1:5′-TGGTAGCTAACCTACTCCAGGAGC-3′, Primer 2:5′-GGTGACTTTG GTGTTCTGCACGAC-3′, and Primer 3:5′-ACTGTGGGCATTCCGTGTACC-3′. A total of 275 mice were used throughout the study. *Gpr56* KO and wild-type (WT) mice were assigned randomly to either the sham or traumatic brain injury group using a lottery box, with 5 or 6 mice per group for most experiments. For the neurological behavioral tests, however, each group consisted of 10 mice to ensure a relatively uniform injury severity and to compare neurological impairments among the different treatment groups. All sample sizes were estimated prior to selection. The groups included: (1) WT group, (2) *Gpr56* KO group, (3) TBI + WT group, and (4) TBI+*Gpr56* KO group. To explore the underlying mechanism of GPR56 targeted CCR5 in TBI, comparable groups were established to receive treatment with either a vehicle or maraviroc (CCR5 antagonist) following TBI: (1) TBI + WT+Vehicle group, (2) TBI+*Gpr56* KO+Vehicle group, (3) TBI+*Gpr56* KO+Maraviroc group. All evaluations were carried out by unbiased investigators who were unaware of the protocols and the types of mice involved. Maraviroc (Selleck Chemicals LLC, Houston, TX, USA) was solubilized in a solution containing 5% dimethyl sulfoxide, 40% polyethylene glycol 300, and 5% Tween 80 in saline. A 20 mg/kg dose of maraviroc [29, 30] was intraperitoneally administered 1 hour after TBI, and the treatment was continued daily for three days. The vehicle group received an equivalent mixture via intraperitoneal injection after TBI. The data were collected by investigators who were blinded to the experimental design, conditions, and treatments in all experiments.

### Murine model of traumatic brain injury

*Gpr56* knockout (KO) and littermate wild-type (WT) mice (males, aged 8–12 weeks, weighing 23–30 g) underwent experimental traumatic brain injury induced by a controlled cortical impact (CCI) device (eCCI-6.3 device, Custom Design & Fabrication, Inc., Sandston, VA, USA) as described previously [31]. In brief, mice were secured to a stereotactic frame (RWD Life Science Co., Ltd., Shenzhen, China) following anesthesia induced by 4% isoflurane, with maintenance at 1.5–2% isoflurane throughout the procedure. A 3.0-mm-diameter opening was created at the midpoint between bregma and lambda, lateral to the sagittal suture on the right parietal bone. The removal of the skull cap was performed delicately to ensure the integrity of the dura. The CCI device was utilized to induce a moderate TBI with the following parameters: a depth of 1.8 mm, velocity of 4.5 m/s, and a duration of 200 ms. Following the TBI procedure, the scalp was promptly closed using 6-0 silk sutures. Each mouse was then placed on a warming pad for anesthesia recovery, subcutaneously administered 5 mg/kg of carprofen (Cayman Chemical, Ann Arbor, MI, USA) and subsequently housed individually. All mice were monitored carefully for at least 4 h after TBI and then daily. Mice in the sham group underwent anesthesia and skull cap removal without CCI induction.

### Modified neurological severity score (mNSS)

The mNSS, which encompasses a combination of motor, sensory, reflex, and balance assessments [32], was used to assess neurological impairments on days 1, 3, 5, 7, and 14 following TBI. The mNSS evaluates neurological function on a scale ranging from 0 to 18. A higher composite score indicates more severe functional impairment.

### Rotarod test

Motor coordination and balance changes in mice were evaluated utilizing a rotarod apparatus (YLS-4C, Beijing) on days 1, 3, 5, 7, and 14 following TBI, as previously described [33]. Before the experiment, mice underwent training on the rotarod at a constant speed of 5–10 RPM for 300 s. During each evaluation day, the rotarod was operated with a consistent accelerating speed, ranging from 5 to 40 RPM, lasting for 300 s, and repeated three times. The latency to fall for each mouse was recorded and then averaged.

### Morris water maze test (MWM)

From 15 to 20 days post-TBI, the mice were subjected to the Morris water maze test [33], which comprises 5 days of training and 1 day of testing. During training (15–19 days), the mice were tasked with finding a hidden platform within 90 s and to stay on it for 15 s. If unsuccessful, the mice were gently guided to the platform and allowed to stay there for 15 s, with a recorded latency of 90 s. Training occurred four times daily, from the first to the fourth quadrant. On the 20th day, the test was conducted without the platform, and the mouse was placed on the opposite side of the platform quadrant. Video tracking (EthoVision XT 13, Noldus Information Technology) recorded and analyzed latency to reach the platform, platform crossings, and swimming traces.

### Brain water content MRI scan

The brain water content was assessed 72 hours after TBI using the wet brain weight/dry brain weight method, as described previously [33, 34]. After the mice were deeply anesthetized, the brains were extracted and perfused with phosphate-buffered saline (PBS). Wet weight (WW) was measured using an electronic analytical balance. Dry weight (DW) was determined similarly after the brains were dried at 100 °C for 24 hours. The brain water content (%) was calculated as (WW-DW)/WW × 100%.

Three days post-TBI, T2-weighted imaging was conducted using a 9.4 T high-field MRI scanner (BioSpec 94/30 USR; Bruker, Billerica, MA, USA) with a slice thickness of 800 µm. Following MR scanning, necrosis areas were quantified based on the T2 images. The corresponding region of interest (ROI) was manually delineated using the RadiAnt DICOM Viewer (Medixant, Poznan, Poland). The total contusion volume and edema area were determined by summing the ROI areas multiplied by the thickness in each scanning plane [35, 36].

### Blood-brain barrier damage

Evans blue (EB) extravasation was employed to evaluate blood-brain barrier (BBB) permeability as described previously [37]. Briefly, the tail vein was used to deliver EB solution (4 ml/kg, 2% in saline; E2129; Sigma Aldrich), which was subsequently allowed to cycle for two hours. Following perfusion with cold PBS, brains were collected for initial imaging and assessment of Evans Blue (EB) extravasation. Subsequently, brain tissues were sectioned into pieces within tubes. Each tube received 1 ml of formamide, and the samples were extracted in a 60 °C water bath for 24 hours. After extraction, the tubes underwent centrifugation at room temperature with a force of 4,000 g for 15 minutes, and 200 µl of the supernatant was added to a 96-well plate. A calibration curve was created by diluting Evans Blue (EB) with formamide in the range of 25.6–0.4 µg/ml. The OD value for each well was measured using absorbance spectroscopy at 610 nm, and the EB concentration in each supernatant was calculated.

### Measurement of cytokines and chemokines

130 mg of brain tissue from the perilesional area was harvested three days following TBI. The brain tissues were used to perform Luminex assay measuring the concentrations of cytokines and chemokines using a Bio-Plex Pro Mouse Cytokine 21-Plex Assay Panel and a Bio-Plex 200 system (Bio-Rad, Hercules, CA, USA) according to the manufacturer’s instructions.

### Western blot

A Western blot was performed according to established protocols [31] using protein extracted from the TBI area of the brain tissue. Polyvinylidene difluoride (PVDF) membranes (Millipore) were blocked with 5% skim milk and incubated overnight at 4 °C with the following primary antibodies: rabbit anti-ZO-1 (1:1000, 61-7300; Thermo Fisher Scientific), mouse anti-claudin-5 (1:1000, 35-2500; Thermo Fisher Scientific), rabbit anti-occludin (1:1000, 27260-1-AP; Proteintech), rabbit anti-cleaved caspase-3 (1:1000, A2156, ABclonal), mouse anti-Bcl2 (1:1000, A20777, ABclonal), mouse anti- P62 (1:1000, A19700, ABclonal), mouse anti-Beclin-1 (1:1000, A22361, ABclonal), rabbit anti-LC3 (1:1000, 3868, Cell Signaling Technology), rabbit anti-GPR56 (1:1000, MABN310, Sigma‒Aldrich), and mouse anti-β-actin (1:1000, TA-09, ZSGB-BIO). The membranes were subsequently incubated with secondary antibodies for 1 hour. The blots were visualized with a ChemiDoc imaging system (Bio-Rad, Hercules, CA, USA) and quantified using ImageJ software.

### Bulk RNA-seq analysis (Transcriptomic profiling)

Three days after TBI, 130-mg brain tissues surrounding the injured cortex were collected and snap-frozen in liquid nitrogen for transcriptome sequencing (5 mice in each group). Total RNA was isolated using TRIzol reagent (Invitrogen, Carlsbad, CA, USA), and RNA libraries were sequenced on an Illumina HiSeq™ 2500/4000 instrument (Gene Denovo Biotechnology Co., Ltd.). Clean reads were generated by removing raw reads containing adapters, low-quality reads, and reads with >10% unknown bases. All clean reads were mapped to genome and gene reference sequences to determine the comparison ratio. Gene expression levels were normalized and standardized using fragments per kilobase of transcript per million mapped reads (FPKM) values. Differentially expressed genes (DEGs) were identified based on edgeR’s general filtering criteria ( | Fold Change | ≥ 1.5, FDR < 0.05) [38, 39]. The expression profiles of DEGs were mapped to Gene Ontology (GO) and KEGG databases to annotate their potential biological processes and metabolic pathways. Statistical analyses were performed using Omicsmart (https://www.omicsmart.com), an interactive online platform for bioinformatics analysis.

### Flow cytometry

Brain tissues three days after TBI were collected and made into single cell suspensions as described in the literature [40]. Briefly, brains were extracted, and the left forebrains were minced into 2 mm pieces in 2.5 ml ice-cold HBSS (without Ca^2+^ and Mg^2+^), containing papain and DNase. The tissue was incubated at 37 °C for 15 min, triturated, and further dissociated for another 15 min. After mixing with ice-cold HBSS+ (0.5% BSA, 2 mM EDTA), samples were centrifuged at 310 g at 4 °C for 5 min. The pellet was resuspended in 1 ml HBSS + , transferred to a chilled tube, and triturated. Cells were centrifuged at 100 g for 15 s, and the supernatant was collected; this was repeated until most cells were dissociated. Cells were filtered through a 40-µm strainer, centrifuged, resuspended in 6 ml of 20% Percoll in HBSS, and centrifuged again at 310 g at 4 °C for 20 min. The myelin layer and supernatant were removed, and the pellet was washed with 3 ml HBSS + . Finally, single cell suspensions were transferred to a FACS tube, centrifuged, and incubated at 4 °C in 50 µl HBSS+ with 1 µl Mouse BD Fc Block for 5 min. Single cell suspensions were stained with fluorescently labeled antibodies: CD45-FITC (1:1000, 157607, Biolegend), NK1.1-PE (1:1000, 156503, Biolegend), CD3-PerCP-Cy5 (1:1000, 100217, Biolegend), Ly6G-PE-Cy7 (1:1000, 127617, Biolegend), CD11b-APC (1:1000, 101211, Biolegend), F4/80-Pacific (1:1000, 123123, Biolegend), CD19-APC-Cy7 (1:1000, 152411, Biolegend), ACSA-2-PE (1:1000, 130-123-284, Miltenyi) at designed combinations. The staining processes were carried out according to the manufacturer’s guidelines. Samples were stained and examined using a FACSAria III flow cytometer (BD Biosciences). Subsequent data analyses were performed with FlowJo 7.6.

### Immunofluorescence

For brain tissues, mice after being anesthetized deeply after TBI, were transcardially perfused with ice-cold PBS followed by whole brains removed quickly and fixed in 4% paraformaldehyde (PFA) at 4 °C for 24 h. The brains were removed and sliced into 8-μm-thick coronal sections using a cryostat (Leica, Model CM1950, Germany). The sections were washed with PBS containing 3% bovine serum albumin, 0.2% Triton, and 0.05% Tween 20 for 1.5 hours at room temperature. After that, the sections were incubated with primary antibodies overnight at 4 °C and then incubated with secondary antibodies for 2 hours at room temperature. The primary antibodies used were as follows: goat anti-Iba-1 (1:200, ab5076, Abcam), rabbit anti-NeuN (1:500, ab177487, Abcam), rabbit anti-LC3 (1:500, 3868, Cell Signaling Technology). After staining, the brain tissue sections were observed using an inverted fluorescence microscope (Olympus, IX73, Japan). For each brain region of interest, at least three non-overlapping fields were imaged per section, with a minimum of three sections per animal. The total number of TUNEL-positive cells and NeuN-positive neurons were quantified using the ImageJ software. The percentage of TUNEL-positive neurons was calculated by dividing the number of TUNEL and NeuN double-positive cells by the total number of NeuN-positive cells and multiplying the result by 100. Quantification for the percentage of LC3 positive neurons follows the same process.

For cells, approximately 1.0–7.0 × 10^4^ cells were cultured on sterile coverslips, treated for 18 hours, and fixed in freshly prepared 4.0% PFA for 30 minutes. Thereafter, the processed cells were also washed with PBS containing 3% bovine serum albumin, 0.2% Triton, and 0.05% Tween 20 for 1.5 hours at room temperature. Subsequently, the cells were subjected to overnight incubation with primary antibodies at 4 °C, followed by a 2-hour incubation with secondary antibodies at room temperature. The primary antibodies used were as follows: rabbit anti-LC3 (1:500, 3868, Cell Signaling Technology), goat anti-CCL3 (1:200, AF-450-NA, R&D Systems), rabbit anti-CCL4 (1:200, ab45690, Abcam), and rabbit anti-CCL5 (1:500, A14192, ABclonal). The stained cells were mounted with coverslips, and images were captured using an inverted fluorescence microscope (Olympus, IX73, Japan). For each experimental condition, at least 3 random fields were imaged. The images were then analyzed using the ImageJ software. The fluorescence intensity of the target proteins was quantified by measuring the mean gray value within the region of interest (ROI) for each cell. The ROI was defined by adjusting the threshold to cover all cells. The mean gray value of each cell was defined as cell fluorescence intensity.

### Establishment of mouse primary microglia

Primary mixed glial cultures were derived from male and female C57BL/6 mouse pups aged 1 to 3 days. In brief, the brains were extracted and placed in ice-cold Hank’s Balanced Salt Solution (HBSS), where the meninges were carefully removed, and the cerebral cortices were isolated. The cells were mechanically dissociated and seeded in 75 cm flasks containing cultured in high-glucose Dulbecco’s modified Eagle’s medium (DMEM, Gibco) cultured with 10% fetal bovine serum (FBS, Sigma-Aldrich) and 1% penicillin-streptomycin (Solarbio). The cultures were maintained at 37 °C in a humidified incubator with 5% CO2. The media was replaced after 24 hours and then every 7 days. Between days 11 and 14, microglia were observed on the astrocyte layer. The microglia were gently detached by manual shaking and subsequently seeded on 6-well plates.

### Transfection of siRNA

For siRNA transfection, siRNAs were synthesized by Sangon Biotech. When the primary microglia/Bv2 microglia reached 70–90% confluence in 6-well plates, GPR56 siRNA (siGPR56) or negative control siRNA (siNC) was transfected into the cells with Lipofectamine 2000 (Solarbio). The sequences of the siRNAs used were as follows: siGPR56-2178 (sense: 5′-CUUCAGCAUCAUAACUUCCUUTT-3′, antisense: 5′-AAGGAAGUUAUGAUGCUGAAGTT-3′); siGPR56-404 (sense: 5′-CCCUCCACUAUGAUCAAUCUUTT-3′, antisense: 5′-AAGAUUGAUCAUAGUGGAGGGTT-3′); siGPR56-1221 (sense: 5′-GCAGAACACCAAAGUCACCAATT-3′, antisense: 5′-UUGGUGACUUUGGUGUUCUGCTT-3′); siNC (sense: 5′-UUCUCCGAACGUGUCACGUTT-3′, antisense: 5′-ACGUGACACGUUCGGAGAATT-3′).

### Cell culture, establishment of a microglia scratch-injury model, drug administration and creation of experimental groups

Primary microglia and Bv2 microglial cells (China Infrastructure of Cell Line Resources, Beijing, China) were cultured in high-glucose Dulbecco’s modified Eagle’s medium (DMEM, Gibco) supplemented with 10% fetal bovine serum (FBS, Sigma‒Aldrich) and 1% penicillin‒streptomycin (Solarbio) in a 5% CO2 incubator at 37 °C. To simulate the trauma model and activation of microglia in vitro, a scratch injury was generated as previously described [41–43]. To assess the effectiveness of siRNAs in silencing, primary microglia/Bv2 microglial cells were divided into four groups: (1) cells transfected with siNC (siNC), (2) cells transfected with siGPR56-2178 (siGPR56-2178), (3) cells transfected with siGPR56-404 (siGPR56-404), and (4) cells transfected with siGPR56-1221 (siGPR56-1221). The primary microglia/Bv2 microglial cells were further categorized into four groups: (1) cells transfected with siNC (siNC), (2) cells transfected with siGPR56-404 (siGPR56-404), (3) cells transfected with siNC and subjected to scratch injury (Injury+siNC), and (4) cells transfected with siGPR56-404 and subjected to scratch injury (Injury+siGPR56-404).

### RNA isolation and quantitative real-time polymerase chain reaction (qRT-PCR)

After extraction using TRIzol reagent (Invitrogen), 1 µg of RNA was used to synthesize cDNA using the Moloney-Murine Leukemia Virus Reverse Transcriptase (TransGen Biotech) according to the manufacturer’s instructions. Analysis was performed in triplicate using the SYBR Green reaction mix (Takara) on QuantStudio 3 Real-Time PCR system (Thermo Fisher Scientific). The primer sequences used for qRT-PCR were as follows: GPR56 (forward: 5′-CTGCGGCAGATGGTCTACTTC-3′, reverse: 5′-CCACACAAAGATGTGAGGCTC-3′); CCL3 (forward: 5′- TGTACCATGACACTCTGCAAC-3′, reverse: 5′-CAACGATGAATTGGCGTGGAA-3′); CCL4 (forward: 5′-TTCCTGCTGTTTCTCTTACACCT-3′, reverse: 5′- CTGTCTGCCTCTTTTGGTCAG-3′); CCL5 (forward: 5′- GCTGCTTTGCCTACCTCTCC-3′, reverse: 5′- TCGAGTGACAAACACGACTGC-3′); GAPDH (forward: 5′-AGGTCGGTGTGAACGGATTTG-3′, reverse: 5′-GGGGTCGTTGATGGCAACA-3′).

### Enzyme-linked immunosorbent assay

The concentrations of CCL3/4/5 released by microglial cells in different groups were assessed and quantified using enzyme-linked immunosorbent assay (ELISA) kits (all obtained from Elabscience) following the guidelines provided by the manufacturer.

### TUNEL staining

Apoptotic cells were detected using the TUNEL assay kit (G3250, Promega) following the manufacturer’s instructions three days after TBI. Cortical neurons were labeled with NeuN immunofluorescence staining. The percentage of TUNEL-positive neurons was analyzed using a fluorescence microscope (Olympus, IX73, Japan) and ImageJ software.

### Nissl staining

Mouse brain tissue was fixed using 4% PFA and subsequently dehydrated in a graded series of ethanol solutions. The paraffin-embedded brain tissues were then sectioned into 8-μm slices for Nissl staining (G1432, Solarbio). Nissl staining was carried out following established protocols [44]. Subsequently, the sections were imaged under a light microscope (Olympus, IX73, Japan).

### Statistical analysis

All the statistical analyses were performed using SPSS 26.0 software (IBM Corporation). The data are presented as the mean ± standard error of the mean (SEM). The Shapiro-Wilk test was used to assess the normality of the data distribution. In order to ensure that the concentration of the cytokine values in the Luminex experiment follow a normal distribution, logarithmic function was used. Differences between two groups were analyzed by an unpaired two-tailed Student’s *t* test. One-way or two-way analysis of variance (ANOVA) with Tukey’s post hoc test was performed for comparisons among multiple groups. *p* < 0.05 indicated a statistically significant difference between experimental groups.

## Results

### Deleting *Gpr56* exacerbates motor and cognitive impairment in TBI mice

**Fig. 1:**
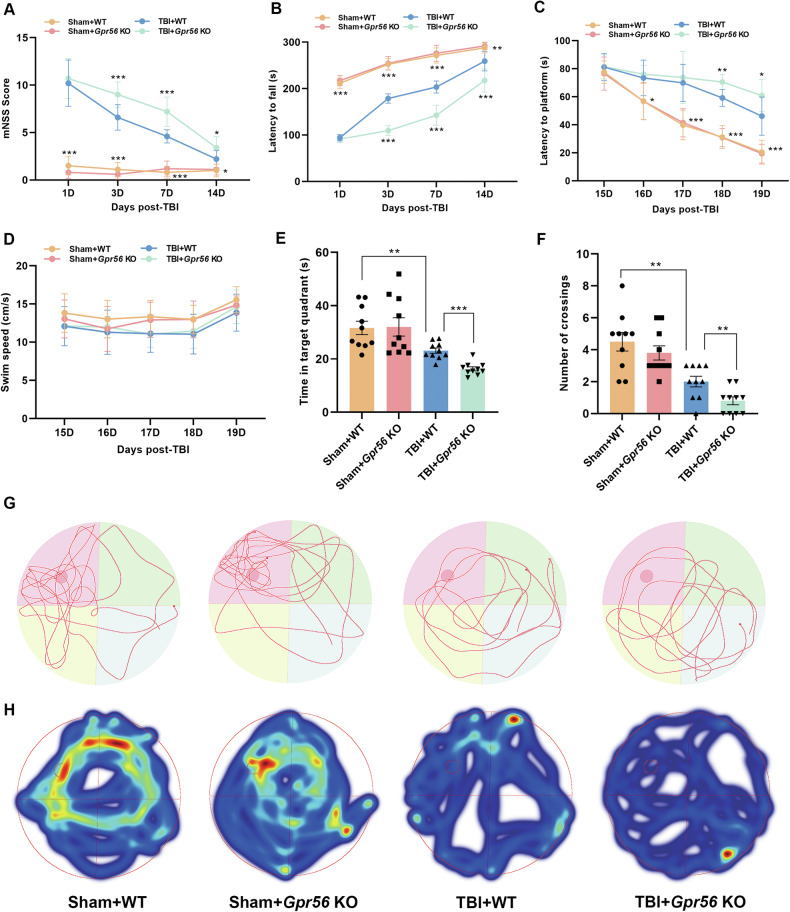
Deletion of *Gpr56* aggravates neurological function deficits in mice after TBI. Neurological performance was assessed by the (**A**) mNSS score, (**B**) rotarod tests, and (**C-F**) MWM tests. Representative track sheets during the probe trial (**G**) and heatmaps of swimming traces (**H**) at 20 days post TBI of MWM tests. *n* = 10 mice per group. One-way analysis of variance followed by Tukey’s post hoc test was used. Data are shown as the mean ± SEM. *p < 0.05, **p < 0.01, ***p < 0.001.

The mNSS tests, rotarod tests and MWM tests were performed to assess the neurological function in *Gpr56* KO and WT mice followed by CCI or sham surgery. The results showed no significant difference between *Gpr56* KO and WT mice under sham conditions. The motor function was impaired in WT mice 1 day after TBI, and loss of *Gpr56* further exacerbated motor deficits at 3–14 d after TBI in comparison with WT mice after TBI (Fig. 1A, B). The MWM tests showed similar results, with *Gpr56* deletion significantly worsening TBI-induced defcits in short-term and spatial memory (Fig. 1C-H). These data demonstrate that deletion of *Gpr56* aggravates motor and cognitive function in mice after TBI.

### Deletion of *Gpr56* aggravates brain tissue loss and BBB damage in TBI mice

**Fig. 2:**
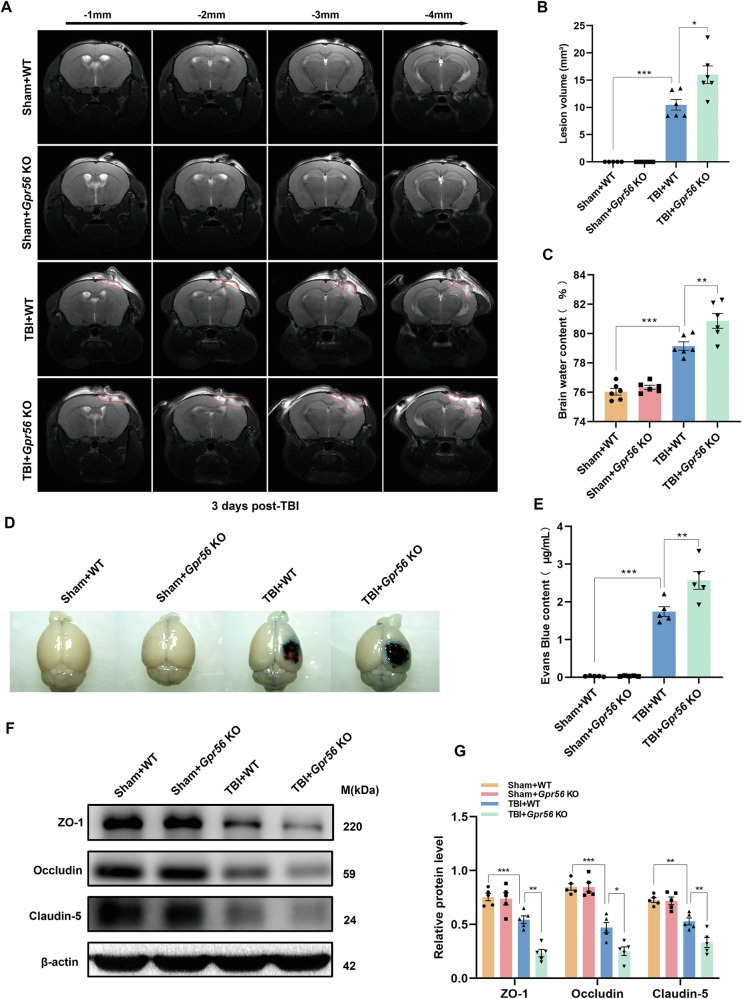
Deletion of *Gpr56* aggravates brain tissue loss and BBB damage in mice three days following TBI. MRI T2WI sequences of the coronal planes showed the lesion area. Coronal T2WIs of the area -1 mm to -4 mm from the same scanning level were acquired. (**A**) Representative T2WIs of each group were taken on day three after TBI. (**B**) Quantification of the lesion volume in different groups. *n* = 6 mice per group. (**C**) Quantification of brain water content. *n* = 6 mice per group. (**D**) Representative images of the EB extravasation assay. Scale bar = 0.5 cm. (**E**) Quantification of EB concentration for each group. *n* = 5 mice per group. (**F**) Representative Western blots of ZO-1, Occludin, and Claudin-5. (**G**) Quantification of relative protein expression normalized to the optical density of β-actin. *n* = 5 mice per group. One-way analysis of variance followed by Tukey’s post hoc test was used. All data are shown as mean ± SEM. *p < 0.05, **p < 0.01, ***p < 0.001.

The lesion volume, brain edema, and BBB damage were evaluated by multiparametric MRI, brain water content, EB extravasation assay, and Western blot in mice three days following TBI. There was no sign of lesion in *Gpr56* KO and WT mice under sham conditions (Fig. 2A, B). Compared with WT controls, *Gpr56* KO mice exhibited a larger lesion volume (Fig. 2A-B), suggesting an increased gross tissue loss after TBI. No significant difference in brain water content was observed between the KO and WT mice under sham conditions, while the brain water content was increased in TBI mice (Fig. 2C). Meanwhile, compared with the WT controls, the *Gpr56* KO mice showed a higher brain water content, implying that brain edema induced by TBI was aggravated in the absence of *Gpr56* (Fig. 2C). Furthermore, the EB extravasation assay showed that TBI induced significant BBB damage in the peri-lesional compared with the sham controls, and the deletion of *Gpr56* increased BBB damage in the peri-lesional (Fig. 2D, E). Tight junction proteins are essential for maintaining the BBB. Therefore, we quantify tight junction protein expression (ZO-1, Occludin, and Claudin-5) by Western blot on brain tissues three days following TBI. The tight junction protein expression levels were decreased three days following TBI compared with the sham controls (Fig. 2F, G). Deleting *Gpr56* led to the reduction of the protein levels of ZO-1, Occludin and Claudin-5 upon TBI (Fig. 2F, G) compared with the WT controls. Taken together, these results indicate that deletion of *Gpr56* aggravates brain tissue loss and BBB damage after TBI in mice.

### *Gpr56* deletion is crucial for the immune response following TBI

**Fig. 3:**
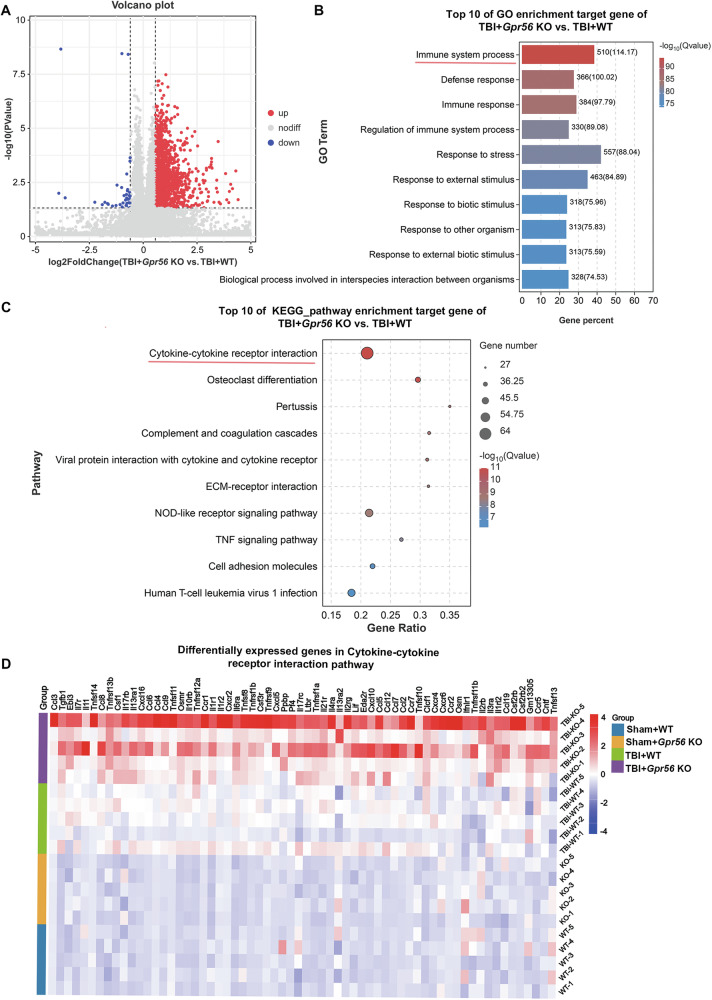
Bioinformatics analysis of the RNA-seq results. **A** Volcano plot showed differentially expressed genes (DEGs) in the TBI+*Gpr56* KO group compared with the TBI + WT group ( | Fold Change | ≥ 1.5, FDR < 0.05). **B** Top 10 GO pathway enrichment analysis of target genes in TBI+*Gpr56* KO and TBI + WT groups. The red line indicates the Immune system process. **C** Top 10 KEGG pathway enrichment analysis of target genes in TBI+*Gpr56* KO and TBI + WT groups. The red line indicates the Cytokines and cytokine receptor interaction pathway. **D** Heatmap of differentially expressed genes in Cytokines and cytokine receptor interaction pathway.

We used bulk transcriptome analysis to identify the different mRNA expression levels between WT and *Gpr56* KO mice three days following TBI. Subsequently, 1,374 differentially expressed genes (DEGs) were screened out by |Fold Change | ≥ 1.5, among which 1,332 genes were highly expressed in *Gpr56* KO mice, and 32 genes were highly expressed in WT mice three days following TBI (Fig. 3A). Gene Ontology (GO) and Kyoto Encyclopedia of Genes and Genomes (KEGG) were used to sift further the significant DEGs between WT and GPR56 KO mice three days following TBI. As shown in Fig. 3B, the top 10 GO terms, classified by -log10(Q value), were significantly enriched in DEGs compared to the genome background Gene Ontology, which is an international standardized gene functional classification system indicating that differentially expressed genes played an essential role in the Immune system process. Furthermore, the KEGG enrichment analysis displayed the classification of the top 10 pathways, revealing that these differentially expressed genes were mainly concentrated in Cytokines and cytokine receptor interaction pathway (Fig. 3C). Next, differentially expressed genes in Cytokines and cytokine receptor interaction pathways were analyzed by heatmap, which showed significant changes in the expression level of chemokines (Fig. 3D), such as CCL4, CCL5, CXCL5, etc. Collectively, through the above analysis, we suggest that *Gpr56* deletion plays a crucial role in the immune response after TBI.

### Deletion of *Gpr56* regulates the release of related chemokines from immune cells in mice three days following TBI

**Fig. 4:**
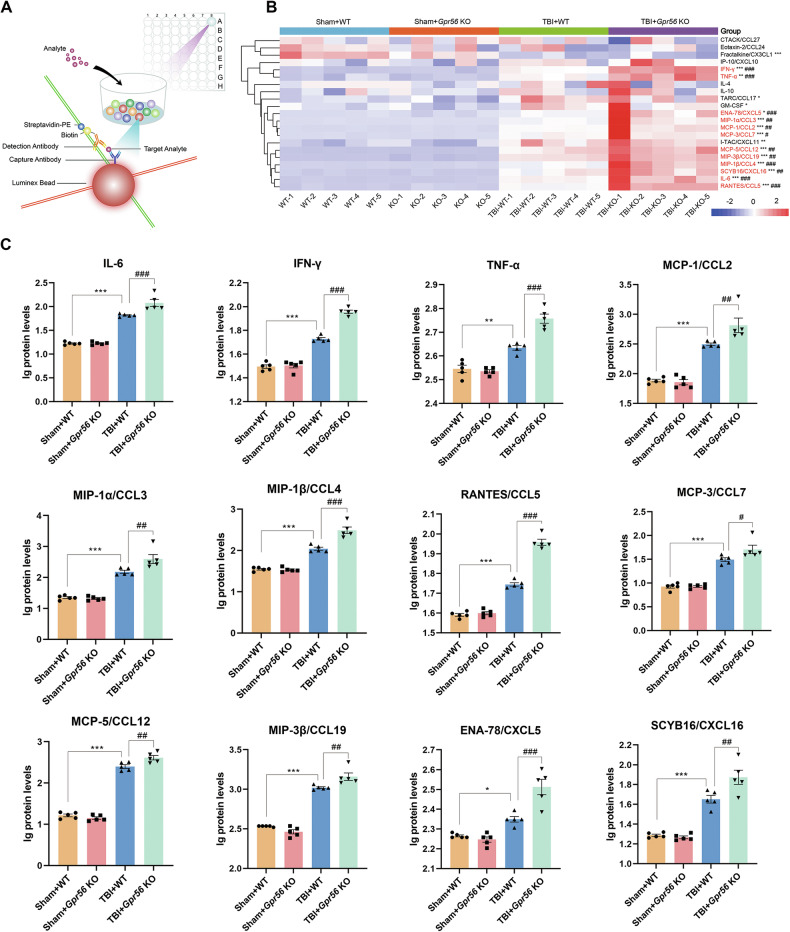
Deleting *Gpr56* regulates the release of chemokines from immune cells in mice three days following TBI. **A** The working model of Luminex assay. **B** Heat map from all Bio-Plex Pro Mouse Chemokine Panel data features 21 magnetic bead-based immunoassays in experimental groups. **C** Quantitative analysis showed significantly increased chemokines in the brains of *Gpr56* KO mice compared to WT controls three days after TBI. *n* = 5 mice per group. One-way analysis of variance followed by Tukey’s post hoc test was used. All data are shown as mean ± SEM. *p < 0.05, **p < 0.01, ***p < 0.001 versus Sham+WT group. ^#^*p* < 0.05, ^##^*p* < 0.01, ^###^*p* < 0.001 versus TBI+*Gpr56* KO group.

Luminex assay based on the principles of microspheres and flow cytometry (Fig. 4A) was used to estimate the protein expression levels of cytokines/chemokines, which were detected by the Bio-Plex Pro Mouse Cytokine Panel featuring 31 magnetic bead-based immunoassays. Our data demonstrated that 16 inflammatory cytokines/chemokines including IL-6, TNF-α, IFN-γ, GM-CSF, CCL2, CCL3, CCL4, CCL5, CCL7, CCL12, CCL17, CCL19, CXCL5, CXCL11, CXCL16, CX3CL were significantly elevated in peri-lesional brain regions at three days after TBI when compared with sham controls (Fig. 4B). Interestingly, deletion of *Gpr56* further increased the protein levels of 12 proinflammatory mediators including IL-6, TNF-α, IFN-γ, CCL2, CCL3, CCL4, CCL5, CCL7, CCL12, CCL19, CXCL5, CXCL16 in peri-lesional brain regions at 3 d after TBI (Fig. 4C). These findings suggest that the GPR56 exerts its protective function in the setting of TBI in part through suppressing pro-inflammatory cytokines/chemokine production directly or indirectly.

### *Gpr56* deletion promotes peripheral immune cell infiltration in mice three days following TBI

**Fig. 5:**
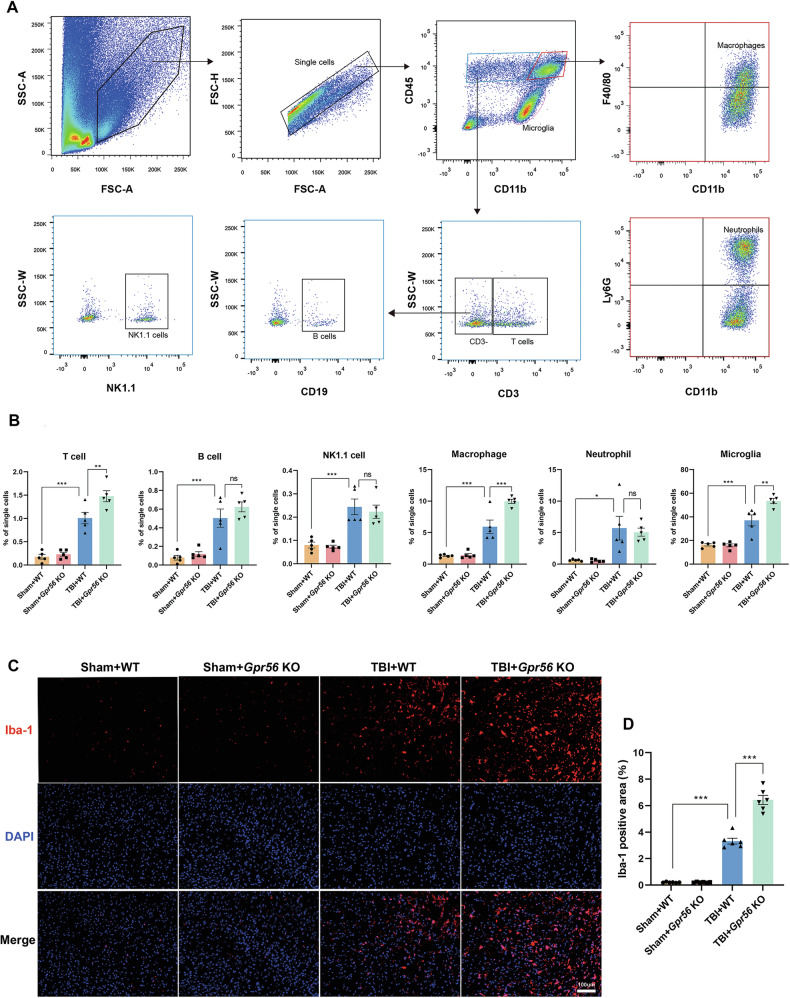
*Gpr56* deletion promotes peripheral immune cell infiltration in mice three days following TBI. **A** Flow cytometry examination of circulating immune cells in the brain three days after TBI. **B** Quantification of immune cells that have infiltrated the brain. *n* = 6 mice per group. **C** Representative immunofluorescence staining images of Iba-1 in the ipsilateral cerebral cortex three days following TBI. Scale bars: 100 µm. **D** Quantification of the Iba-1 area. *n* = 6 mice per group. One-way analysis of variance followed by Tukey’s post hoc test was used. All data are shown as mean ± SEM. *p < 0.05, **p < 0.01, ***p < 0.001.

Post-trauma BBB disruption and leakage lead to several subsequent pathological processes, including the infiltration of peripheral immune cells. GO analysis revealed that GPR56 plays a vital role in the immune system process in mice three days after TBI. Hence, we profiled immune cells, including T cell, B cell, NK cell, macrophage, neutrophil, and microglia, in the brain three days after TBI using flow cytometry (Fig. 5A). The proportion of detected immune cells increased after TBI versus the sham controls (Fig. 5B). *Gpr56* deletion exacerbated the invasion of T cells, macrophages, and microgliosis in post-trauma brains compared to WT controls in the brains three days after TBI (Fig. 5B). Moreover, Iba-1 immunofluorescence confirmed microgliosis compared to sham controls and in *Gpr56* KO mice further promoted the microgliosis compared to WT controls (Fig. 5C, D) three days after TBI. These findings indicate that deleting *Gpr56* results in increased infiltration of peripheral immune cells, including T cells and macrophages, into the brain and microgliosis following TBI, thereby amplifying cerebral inflammatory responses.

### TBI reduces the GPR56 expression in microglia

**Fig. 6:**
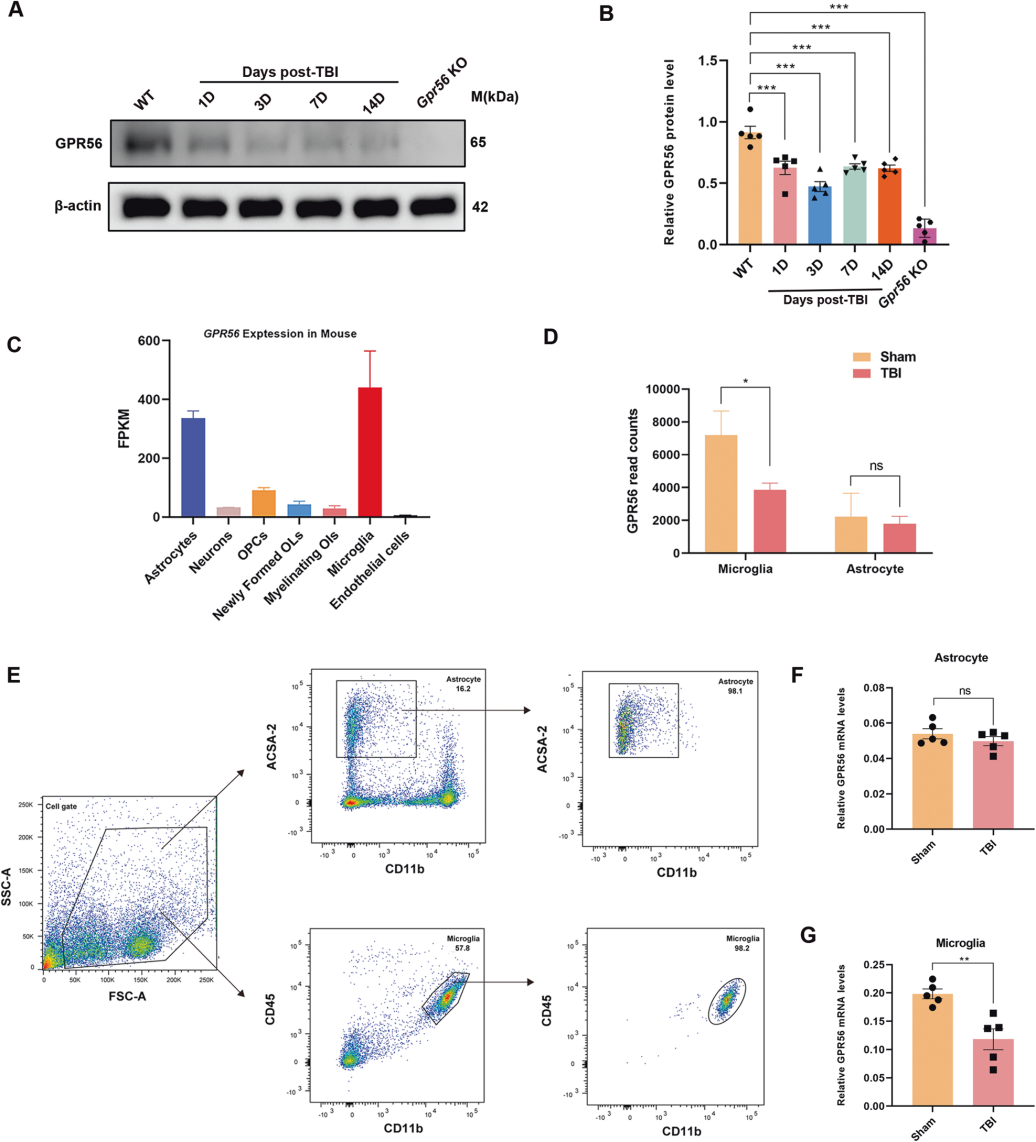
GPR56 expression levels were reduced in microglia after TBI. **A** Representative Western blots of GPR56. **B** Quantification of relative protein expression normalized to the optical density of β-actin. **C** GPR56 expression analysis of the GSE52564 dataset in different brain cell types. **D** GPR56 expression analysis with the GSE167459 dataset in astrocytes and microglia between Sham and TBI group. **E** Isolation of astrocytes and microglia by Flow cytometry. **F** Changes in mRNA expression levels of GPR56 in astrocyte three days following TBI. **G** Changes in mRNA expression levels of GPR56 in microglial three days following TBI. *n* = 5 mice per group. All data are shown as mean ± SEM. *p < 0.05, **p < 0.01, ***p < 0.001.

To further investigate the role of GPR56 in mice after TBI, GPR56 protein expression levels were detected by Western blot after TBI in WT mice. Western blot analysis revealed that GPR56 protein expression levels were decreased at 1 d, 3 d, 7 d and 14 d after TBI (Fig. 6A, B). In order to explore the relationship between GPR56 and microglia in mice after TBI, we analyzed GPR56 mRNA expression in the GSE52564 and GSE167459 datasets downloaded from the NCBI website. GSE52564 dataset analysis revealed that GPR56 was highly expressed in astrocytes and microglia [16, 17] (Fig. 6C). Furthermore, GSE167459 datasets showed that GPR56 was downregulated in microglia in mice three days after TBI while showing no significant difference in astrocytes after TBI through GSE167459 dataset analysis (Fig. 6D). To verify this result, ipsilateral tissues were harvested to performed concurrent microglial and astrocyte isolation by Flow cytometry (Fig. 6E). qRT-PCR analysis showed that there was no significant difference of GPR56 mRNA expression levels in astrocytes three days following TBI (Fig. 6F), whereas GPR56 mRNA expression levels were reduced in microglia three days following TBI (Fig. 6G). Based on these analyses, it appears that GPR56 and microglia function in TBI are strongly connected.

### GPR56 deficiency promotes injury-induced inflammatory response in primary microglia in vitro

**Fig. 7:**
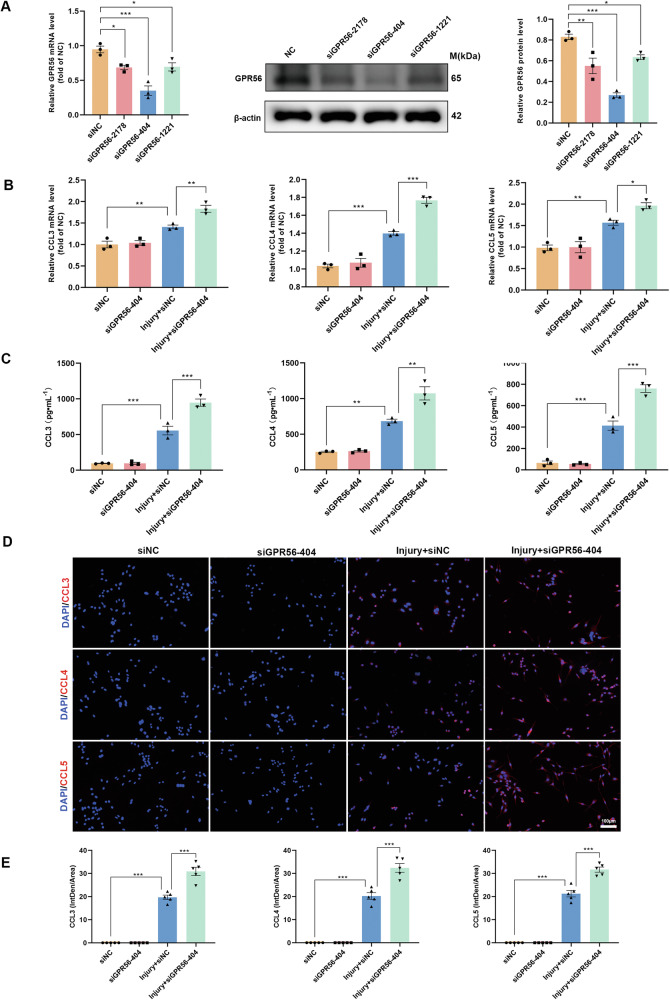
Downregulation of GPR56 in cultured primary microglia promotes chemokine secretion. **A** The qRT-PCR and Western blot analysis revealed the efficiency of GPR56 downregulation in primary microglia by different GPR56 siRNAs in mRNA and protein levels. **B** Changes in the primary microglia mRNA expression levels of pro-inflammatory chemokines CCL3, CCL4, and CCL5 in experimental groups. **C** ELISA results of chemokines CCL3, CCL4, and CCL5 in experimental groups. **D** Representative fluorescence images of chemokines CCL3, CCL4, and CCL5 in primary microglia in experimental groups. Scale bars: 100 µm. **E** Quantitative analysis of CCL3/4/5 fluorescence intensities. The in vitro experiments were repeated at least three times. One-way analysis of variance followed by Tukey’s post hoc test was used. All data are shown as mean ± SEM. *p < 0.05, **p < 0.01, ***p < 0.001.

According to earlier research, neuroinflammatory signaling by microglia is crucial for subsequent degeneration after TBI [45, 46], and activated microglia secrete a large amount of CCL3, CCL4, and CCL5 [47]. Therefore, we examined if GPR56 knockdown regulates the secretion of CCL3, CCL4, and CCL5 by microglia. Firstly, primary microglia were obtained and identified by immunofluorescence (Fig. S2). To achieve this goal, three siRNA sequences were initially designed (siGPR56-2178, siGPR56-404, and siGPR56-1221) for GPR56 knockdown. It was observed that transfection with these three siRNAs resulted in a reduction in both mRNA and protein expression levels of GPR56 in primary microglia, with siGPR56-404 showing a particularly significant effect (Fig. 7A). Further analysis through qRT-PCR assays and ELISA revealed no significant difference in CCL3, CCL4, and CCL5 mRNA expression levels between siNC (negative control siRNA) and siGPR56-404 treated primary microglia without scratch-injury. In comparison, scratch injury increased the expression levels of CCL3, CCL4, and CCL5 in primary microglia, while GPR56 knockdown exacerbated this process (Fig. 7B, C). Consistent with these findings, immunofluorescence analysis demonstrated that scratch injury increased the density of CCL3/4/5, and GPR56 knockdown further enhanced the density in primary microglia. These results were further confirmed in Bv2 microglial cells (Fig. S3). These data demonstrate that GPR56 knockdown promotes microglia secretion of CCL3/4/5 after injury.

### CCR5 inhibition promotes autophagy and reverses neuronal apoptosis caused by *Gpr56* deletion in TBI mice

**Fig. 8:**
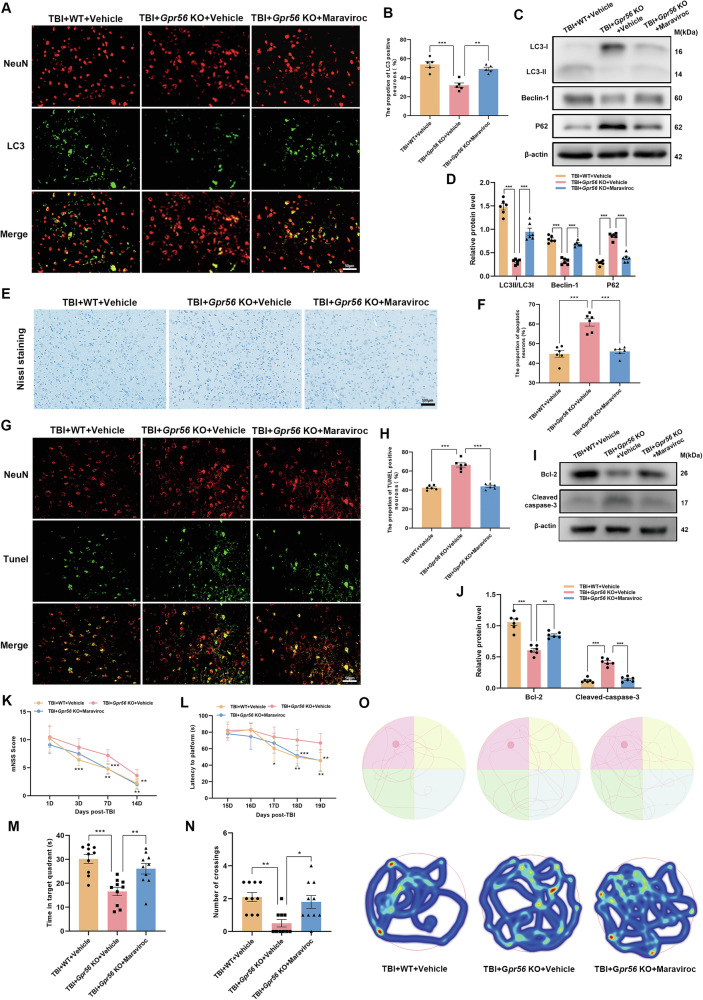
CCR5 antagonist rescues the worsening neurological function deficits induced by *Gpr56* deletion three days following TBI. **A** Representative images of LC3-positive neurons in the ipsilateral cerebral cortex three days following TBI. Scale bars: 50 µm. **B** Quantification of LC3-positive neurons. *n* = 6 mice per group. **C** Representative Western blots of LC3-I, LC3-II, Beclin-1, and P62. **D** Quantification of LC3-II/LC3-I, Beclin-1, and P62 protein expression. *n* = 6 mice per group. **E** The representative images of Nissl staining of the ipsilateral cerebral cortex three days following TBI. Scale bars: 100 µm. **F** Quantification of Nissl staining for the apoptotic neurons. *n* = 6 mice per group. **G** Representative images of TUNEL-positive neurons in the ipsilateral cerebral cortex three days following TBI. Scale bars: 50 µm. **H** Quantification of TUNEL-positive neurons. *n* = 6 mice per group. **I** Representative Western blots of Cleaved caspase-3 and Bcl-2. **J** Quantification of Cleaved caspase-3 and Bcl-2 protein expression. *n* = 6 mice per group. Neurological performance (*n* = 10 per group) was assessed by the (**K**) mNSS score and (**L-O**) MWM tests. Representative track sheets during the probe trial and heatmaps of swimming traces (**O**) at 20 days post TBI of MWM tests. One-way analysis of variance followed by Tukey’s post hoc test was used. Data are shown as the mean ± SEM. *p < 0.05, **p < 0.01, ***p < 0.001.

Recent research revealed that microglial-derived CCL3/4/5 activates neuronal CCR5 to suppress neuronal autophagy in Huntington’s disease and tauopathies [47]. Our study displayed that *Gpr56* deletion regulated the release of CCL3, CCL4, and CCL5 in TBI mice (Fig. 4). Additionally, scratch injury boosted the secretion of CCL3, CCL4, and CCL5 in primary microglia, and *Gpr56* knockdown aggravated this phenomenon (Fig. 7). Therefore, we reasoned that *Gpr56* deletion aggravates traumatic brain injury via CCL3/4/5-CCR5 signaling. To test this hypothesis, the *Gpr56* KO mice were treated with maraviroc (an antagonist of CCR5) after TBI. Immunofluorescence and Western bolt were used to detect the markers of autophagy. Compared with the WT group, immunofluorescence revealed a decreasing number of neurons with autophagy and maraviroc treatment reversed this process (Fig. 8A, B). Western blot analyses showed that *Gpr56* deletion decreased the expression of LC3-II/LC3-I and Beclin-1 while increasing the expression of P62 in mice three days following TBI compared to the WT group (Fig. 8A-D), whereas maraviroc treatment reversed this process (Fig. 8C, D). In parallel, Nissl staining experiments showed that compared to the WT group, deletion of the *Gpr56* promoted neuronal apoptosis in mice three days after TBI (Fig. 8E, F). Treatment with Maraviroc rescued the increased neuronal apoptosis caused by *Gpr56* deletion (Fig. 8E, F). TUNEL staining experiments revealed that *Gpr56* deletion increased the number of apoptotic neurons three days after TBI compared to the WT group, and Maraviroc treatment reversed this effect (Fig. 8G, H). Western blot analyses demonstrated that *Gpr56* deletion increased the protein expression level of Cleaved caspase-3 and decreased the protein expression level of Bcl-2 (Fig. 8I, J) in mouse brain tissue three days after TBI, compared to the WT group. Maraviroc treatment reversed the *Gpr56* deletion-induced increase in Cleaved caspase-3 protein expression and decrease in Bcl-2 protein expression in mouse brain tissue three days post-TBI (Fig. 8I, J). The mNSS and MWM test results showed that compared to the WT group, *Gpr56* deletion aggravated the neurological impairment of mice post-traumatic brain injury, and treatment with Maraviroc reversed the process caused by *Gpr56* deletion post-TBI (Fig. 8K-O). These results demonstrate that *Gpr56* deletion restrains neuronal autophagy and aggravates neuronal apoptosis in mice three days following TBI via CCR5 signaling.

## Discussion

GPR56, a member of the adhesion G-protein coupled receptor (aGPCR) family, mediates cell-cell and cell-extracellular interaction [10, 13]. It regulates peripheral inflammatory response through T and NK cells [18]. However, the functional importance of GPR56 in the pathogenesis of brain trauma is not known. The current research is the first to explore the impact of GPR56 deficiency on TBI. We showed that *Gpr56* deletion aggravated motor and cognitive function impairment in mice after TBI, positively correlated with worsened brain tissue loss and increased BBB damage in post-trauma brains. *Gpr56* deletion further promoted the release of inflammatory chemokines to the post-trauma brains. Additionally, *Gpr56* deletion led to microgliosis and T cell and macrophage infiltration to brain parenchyma in TBI mice. GPR56 downregulation promoted CCL3/4/5 secretion from activated microglia. In TBI mice, CCR5 inhibition boosts autophagy and prevents neuronal death induced by *Gpr56* deletion. Additionally, CCR5 inhibition facilitated the recovery of the motor and cognitive function impairment exacerbated by *Gpr56* deletion. Our findings suggest that GPR56 is a protective factor in the pathophysiology of TBI.

Multiple studies have consistently demonstrated that both TBI patients and rodent experimental TBI models frequently exhibit neurobehavioral dysfunction and associated sequelae [6, 48–52]. Our research specifically indicates that the absence of *Gpr56* intensifies motor and cognitive impairment post-TBI, highlighting GPR56’s crucial role in regulating these functions after brain trauma.

Brain edema, a significant secondary injury after TBI, results from blood-brain barrier disruption involving cytotoxic and vasogenic mechanisms [53, 54]. Our study revealed that *Gpr5*6 deletion exacerbated TBI-induced brain edema, as demonstrated by MRI and brain water content, and increased Evan’s blue leakage. Additionally, *Gpr56* deletion worsened vasogenic brain edema by augmenting tight junction protein loss. Loss of microvascular structures is known to contribute to BBB breakdown in various central nervous system injury models [55, 56]. Our experiment found that *Gpr56* deletion decreased tight junction protein expression after TBI, further breaking the BBB.

An RNA-seq analysis was conducted to identify additional genes that might be associated with the effects of *Gpr56* deletion in mice three days following TBI. According to the RNA-seq results, differentially expressed genes in the TBI+*Gpr56* KO group mainly participated in the immune system process and cytokines and cytokine receptor interaction pathway compared with the TBI + WT group. Current studies reported that GPR56 is widely present on the surface of cytotoxic NK cells and T cells in peripheral blood, regulating peripheral inflammatory response [18]. Activation of NK cells lead to a decrease in GPR56 expression on the surface. At the same time, overexpression of GPR56 reduces NK cell cytotoxicity and cytokine production [57], suggesting that GPR56 can inhibit excessive inflammatory response of immune cells.

We found that *Gpr56* deletion exacerbated the upregulation of cytokines/chemokines (IL-6, TNF-α, IFN-γ, CCL2, CCL3, CCL4, CCL5, CCL7, CCL12, CCL19, CXCL5, CXCL16) in protein levels in the central nervous system while promoting peripheral immune cell (T cells, macrophages) infiltration into the brain parenchyma and microgliosis compared to WT controls after TBI. As reported in the literature, a multitude of inflammatory mediators are released into the cerebral parenchyma in response to brain trauma stimuli, along with glial cell activation and peripheral immune cell infiltration [6, 8, 58–60]. Therefore, these results demonstrated that GPR56 is involved in immune regulation and neuroinflammation after traumatic brain injury.

Microglia is the resident immune cell in the CNS, which plays a central role in CNS inflammation [61]. Previous studies in rodent CCI models indicated that microglia were increased in pericontusional brain regions and promoted a pro-inflammatory response after TBI [62–64], consistent with our results. Interestingly, *Gpr56* deletion further promoted this phenomenon. In addition, the analysis of GSE52564 and GSE167459 datasets revealed that GPR56 was highly expressed in astrocytes and microglia and was downregulated in microglia in mice three days after TBI. The above findings suggested a robust correlation between GPR56 and the role of microglia in traumatic brains. An RNA sequencing performed on microglia from aged mice [65], where microglial activation happens concurrently with reduced autophagy in the brain [66, 67], was analyzed by Festa et al. Among the chemokines and cytokines, they discovered that CCL3, CCL4, and CCL5 were the most substantially elevated factors [47]. In addition, Festa et al. reported that activated microglia regulated neuronal autophagy non-cell autonomously via secretion of CCL3, CCL4, and CCL5 in neurodegenerative diseases, including tauopathies and Huntington’s disease [47]. We found similar results that scratch injury promoted the secretion of CCL3/CCL4/CCL5 by microglia, while GPR56 knockdown exacerbated this process. CCL3, CCL4, and CCL5 exhibit strong binding to CCR5, a G protein-coupled receptor (GPCR) typically found in immune cells [68]. Recent studies have revealed the expression of CCR5 in neurons [29, 69–71], and CCL3/4/5 disrupts neuronal autophagy and autophagy substrate clearance via CCR5[47]. Due to the role of CCR5 as an HIV co-receptor, there is increasing interest in the development of pharmacological CCR5 inhibitors [72]. This effort has resulted in the U.S. Food and Drug Administration (FDA) approval of maraviroc, a drug designed to inhibit CCR5 activity selectively [73]. Our research found that *Gpr56* deletion restrained neuronal autophagy and aggravated neuronal apoptosis in mice three days following TBI, while maraviroc partially reversed this process. Similarly, the worsening of neurological function deficits induced by *Gpr56* deletion in mice after TBI was also partially reversed by maraviroc. These results indicate that *Gpr56* deletion exerts neuronal apoptosis and attenuated neuroprotective effects by weakening autophagy after TBI, possibly through the strong binding of CCL3/CCL4/CCL5 to CCR5.

Overall, we demonstrated that *Gpr56* deletion aggravated TBI, and GPR56 regulation of microglia might represent one of the major mechanisms for its protective role in brain trauma. Vizurraga et al. reported that hexahydroquinoline derivatives were a selective agonist for GPR56 [74]. However, due to the lack of a commercial agonist for GPR56, we didn’t activate the GPR56 after TBI to see whether it could reverse the phenotype. Nevertheless, there are some limitations in our study. Firstly, due to the high expression of GPR56 in astrocytes and microglia, microglia-specific deletion of *Gpr56* needs to be established for further study. Secondly, TBI-induced secondary injury is a complicated pathophysiological process, future research examining the interaction of apoptosis, autophagy, and neuroinflammation is needed to clarify the biological mechanisms of GPR56 in TBI-induced neuronal apoptosis. Thirdly, our study didn’t upregulate the expression of GPR56 to observe whether it can improve neurological function after TBI and its molecular protective mechanisms, which need to be further studied in the future.

In conclusion, TBI caused a reduction of GPR56 and *Gpr56* deletion worsened neurological function deficits and BBB damage after TBI through the underlying mechanism of microglial-to-neuronal CCR5 signaling. Increasing the expression of GPR56 or developing a potent agonist may be a new direction for the pharmacological treatment of TBI.

## Supplementary information

Full uncropped Gels and Blots images

Supplemental figures

## Data Availability

The datasets generated and analyzed during the current study are available from the corresponding author upon reasonable request.

## References

[1] Dewan MC, Rattani A, Gupta S, Baticulon RE, Hung YC, Punchak M, et al. Estimating the global incidence of traumatic brain injury. J Neurosurg. 2018;1304:1080–97. 10.3171/2017.10.JNS17352 29701556

[2] Thapa K, Khan H, Singh TG, Kaur A. Traumatic Brain Injury: Mechanistic Insight on Pathophysiology and Potential Therapeutic Targets. J Mol Neurosci. 2021;719:1725–42. 10.1007/s12031-021-01841-7 33956297

[3] Corrigan F, Mander KA, Leonard AV, Vink R. Neurogenic inflammation after traumatic brain injury and its potentiation of classical inflammation. J Neuroinflammation. 2016;131:264. 10.1186/s12974-016-0738-9 PMC5057243 27724914

[4] Nebie O, Carvalho K, Barro L, Delila L, Faivre E, Renn TY, et al. Human platelet lysate biotherapy for traumatic brain injury: preclinical assessment. Brain. 2021;14410:3142–58. 10.1093/brain/awab205 PMC8634089 34086871

[5] Clausen F, Hånell A, Israelsson C, Hedin J, Ebendal T, Mir AK, et al. Neutralization of interleukin-1β reduces cerebral edema and tissue loss and improves late cognitive outcome following traumatic brain injury in mice. Eur J Neurosci. 2011;341:110–23. 10.1111/j.1460-9568.2011.07723.x 21623956

[6] Dixon KJ. Pathophysiology of Traumatic Brain Injury. Phys Med Rehabil Clin N. Am. 2017;282:215–25. 10.1016/j.pmr.2016.12.001 28390509

[7] Vaibhav K, Braun M, Alverson K, Khodadadi H, Kutiyanawalla A, Ward A, et al. Neutrophil extracellular traps exacerbate neurological deficits after traumatic brain injury. Sci Adv. 2020;622:eaax8847. 10.1126/sciadv.aax8847 PMC7259928 32523980

[8] Lozano D, Gonzales-Portillo GS, Acosta S, de la Pena I, Tajiri N, Kaneko Y, et al. Neuroinflammatory responses to traumatic brain injury: etiology, clinical consequences, and therapeutic opportunities. Neuropsychiatr Dis Treat. 2015;11:97–106. 25657582 10.2147/NDT.S65815 PMC4295534

[9] Alves JL, Rato J, Silva V. Why Does Brain Trauma Research Fail? World Neurosurg. 2019;130:115–21. 31284053 10.1016/j.wneu.2019.06.212

[10] Hauser AS, Attwood MM, Rask-Andersen M, Schiöth HB, Gloriam DE. Trends in GPCR drug discovery: new agents, targets and indications. Nat Rev Drug Discov. 2017;1612:829–42. 10.1038/nrd.2017.178 PMC6882681 29075003

[11] Iguchi T, Sakata K, Yoshizaki K, Tago K, Mizuno N, Itoh H. Orphan G protein-coupled receptor GPR56 regulates neural progenitor cell migration via a G alpha 12/13 and Rho pathway. J Biol Chem. 2008;28321:14469–78. 10.1074/jbc.M708919200 18378689

[12] Luo R, Jeong SJ, Jin Z, Strokes N, Li S, Piao X. G protein-coupled receptor 56 and collagen III, a receptor-ligand pair, regulates cortical development and lamination. Proc Natl Acad Sci USA. 2011;10831:12925–30. 10.1073/pnas.1104821108 PMC3150909 21768377

[13] Folts CJ, Giera S, Li T, Piao X. Adhesion G Protein-Coupled Receptors as Drug Targets for Neurological Diseases. Trends Pharmacol Sci. 2019;404:278–93. 10.1016/j.tips.2019.02.003 PMC6433515 30871735

[14] Jeong SJ, Li S, Luo R, Strokes N, Piao X. Loss of Col3a1, the gene for Ehlers-Danlos syndrome type IV, results in neocortical dyslamination. PLoS One. 2012;71:e29767. 10.1371/journal.pone.0029767 PMC3250483 22235340

[15] Jeong SJ, Luo R, Li S, Strokes N, Piao X. Characterization of G protein-coupled receptor 56 protein expression in the mouse developing neocortex. J Comp Neurol. 2012;52013:2930–40. 10.1002/cne.23076 PMC3908671 22351047

[16] Zhang Y, Chen K, Sloan SA, Bennett ML, Scholze AR, O’Keeffe S, et al. An RNA-sequencing transcriptome and splicing database of glia, neurons, and vascular cells of the cerebral cortex. J Neurosci. 2014;3436:11929–47. 10.1523/JNEUROSCI.1860-14.2014 PMC4152602 25186741

[17] Bennett ML, Bennett FC, Liddelow SA, Ajami B, Zamanian JL, Fernhoff NB, et al. New tools for studying microglia in the mouse and human CNS. Proc Natl Acad Sci USA. 2016;11312:E1738–46. 10.1073/pnas.1525528113 PMC4812770 26884166

[18] Peng YM, van de Garde MD, Cheng KF, Baars PA, Remmerswaal EB, van Lier RA, et al. Specific expression of GPR56 by human cytotoxic lymphocytes. J Leukoc Biol. 2011;904:735–40. 10.1189/jlb.0211092 21724806

[19] Chang GW, Hsiao CC, Peng YM, Vieira Braga FA, Kragten NA, Remmerswaal EB, et al. The Adhesion G Protein-Coupled Receptor GPR56/ADGRG1 Is an Inhibitory Receptor on Human NK Cells. Cell Rep. 2016;158:1757–70. 10.1016/j.celrep.2016.04.053 27184850

[20] Bennett FC, Bennett ML, Yaqoob F, Mulinyawe SB, Grant GA, Hayden Gephart M, et al. A Combination of Ontogeny and CNS Environment Establishes Microglial Identity. Neuron. 2018;986:1170–83.e8. 10.1016/j.neuron.2018.05.014 PMC6023731 29861285

[21] Gosselin D, Link VM, Romanoski CE, Fonseca GJ, Eichenfield DZ, Spann NJ, et al. Environment drives selection and function of enhancers controlling tissue-specific macrophage identities. Cell. 2014;1596:1327–40. 10.1016/j.cell.2014.11.023 PMC4364385 25480297

[22] Jeong SJ, Luo R, Singer K, Giera S, Kreidberg J, Kiyozumi D, et al. GPR56 functions together with α3β1 integrin in regulating cerebral cortical development. PLoS One. 2013;87:e68781. 10.1371/journal.pone.0068781 PMC3706371 23874761

[23] Singer K, Luo R, Jeong SJ, Piao X. GPR56 and the developing cerebral cortex: cells, matrix, and neuronal migration. Mol Neurobiol. 2013;471:186–96. 10.1007/s12035-012-8343-0 PMC3538897 23001883

[24] Giera S, Deng Y, Luo R, Ackerman SD, Mogha A, Monk KR, et al. The adhesion G protein-coupled receptor GPR56 is a cell-autonomous regulator of oligodendrocyte development. Nat Commun. 2015;6:6121. 25607655 10.1038/ncomms7121 PMC4302951

[25] Ackerman SD, Garcia C, Piao X, Gutmann DH, Monk KR. The adhesion GPCR Gpr56 regulates oligodendrocyte development via interactions with Gα12/13 and RhoA. Nat Commun. 2015;6:6122. 25607772 10.1038/ncomms7122 PMC4302765

[26] Giera S, Luo R, Ying Y, Ackerman SD, Jeong SJ, Stoveken HM, et al. Microglial transglutaminase-2 drives myelination and myelin repair via GPR56/ADGRG1 in oligodendrocyte precursor cells. Elife. 2018;7:e33385. 10.7554/eLife.33385 PMC5980231 29809138

[27] Li T, Chiou B, Gilman CK, Luo R, Koshi T, Yu D, et al. A splicing isoform of GPR56 mediates microglial synaptic refinement via phosphatidylserine binding. Embo j. 2020;3916:e104136. 10.15252/embj.2019104136 PMC7429740 32452062

[28] Chiou B, Gao C, Giera S, Folts CJ, Kishore P, Yu D, et al. Cell type-specific evaluation of ADGRG1/GPR56 function in developmental central nervous system myelination. Glia. 2021;692:413–23. 10.1002/glia.23906 32902916

[29] Joy MT, Ben Assayag E, Shabashov-Stone D, Liraz-Zaltsman S, Mazzitelli J, Arenas M, et al. CCR5 Is a Therapeutic Target for Recovery after Stroke and Traumatic Brain Injury. Cell. 2019;1765:1143–57.e13. 10.1016/j.cell.2019.01.044 PMC7259116 30794775

[30] Friedman-Levi Y, Liraz-Zaltsman S, Shemesh C, Rosenblatt K, Kesner EL, Gincberg G, et al. Pharmacological blockers of CCR5 and CXCR4 improve recovery after traumatic brain injury. Exp Neurol. 2021;338:113604. 33453212 10.1016/j.expneurol.2021.113604

[31] Liu XL, Sun DD, Zheng MT, Li XT, Niu HH, Zhang L, et al. Maraviroc promotes recovery from traumatic brain injury in mice by suppression of neuroinflammation and activation of neurotoxic reactive astrocytes. Neural Regen Res. 2023;181:141–9. 10.4103/1673-5374.344829 PMC9241405 35799534

[32] Chen J, Li Y, Wang L, Zhang Z, Lu D, Lu M, et al. Therapeutic benefit of intravenous administration of bone marrow stromal cells after cerebral ischemia in rats. Stroke. 2001;324:1005–11. 10.1161/01.str.32.4.1005 11283404

[33] Gao C, Qian Y, Huang J, Wang D, Su W, Wang P, et al. A Three-Day Consecutive Fingolimod Administration Improves Neurological Functions and Modulates Multiple Immune Responses of CCI Mice. Mol Neurobiol. 2017;5410:8348–60. 10.1007/s12035-016-0318-0 27924525

[34] Su Y, Fan W, Ma Z, Wen X, Wang W, Wu Q, et al. Taurine improves functional and histological outcomes and reduces inflammation in traumatic brain injury. Neuroscience. 2014;266:56–65. 24530657 10.1016/j.neuroscience.2014.02.006

[35] Sturiale CL, De Bonis P, Rigante L, Calandrelli R, D’Arrigo S, Pompucci A, et al. Do traumatic brain contusions increase in size after decompressive craniectomy? J Neurotrauma. 2012;2918:2723–6. 10.1089/neu.2012.2556 22873699

[36] Szczygielski J, Hubertus V, Kruchten E, Müller A, Albrecht LF, Mautes AE, et al. Brain Edema Formation and Functional Outcome After Surgical Decompression in Murine Closed Head Injury Are Modulated by Acetazolamide Administration. Front Neurol. 2019;10:273. 30972006 10.3389/fneur.2019.00273 PMC6443632

[37] Hu J, Wang X, Chen X, Fang Y, Chen K, Peng W, et al. Hydroxychloroquine attenuates neuroinflammation following traumatic brain injury by regulating the TLR4/NF-κB signaling pathway. J Neuroinflammation. 2022;191:71. 10.1186/s12974-022-02430-0 PMC8961949 35346242

[38] Love MI, Huber W, Anders S. Moderated estimation of fold change and dispersion for RNA-seq data with DESeq2. Genome Biol. 2014;1512:550. 10.1186/s13059-014-0550-8 PMC4302049 25516281

[39] Robinson MD, McCarthy DJ, Smyth GK. edgeR: a Bioconductor package for differential expression analysis of digital gene expression data. Bioinformatics. 2010;261:139–40. 10.1093/bioinformatics/btp616 PMC2796818 19910308

[40] Todd BP, Chimenti MS, Luo Z, Ferguson PJ, Bassuk AG, Newell EA. Traumatic brain injury results in unique microglial and astrocyte transcriptomes enriched for type I interferon response. J Neuroinflammation. 2021;181:151. 10.1186/s12974-021-02197-w PMC8259035 34225752

[41] Wang H, Song X, Li M, Wang X, Tao Y, Xiya X, et al. The role of TLR4/NF-κB signaling pathway in activated microglia of rats with chronic high intraocular pressure and vitro scratch injury-induced microglia. Int Immunopharmacol. 2020;83:106395. 32199351 10.1016/j.intimp.2020.106395

[42] Han Z, Chen F, Ge X, Tan J, Lei P, Zhang J. miR-21 alleviated apoptosis of cortical neurons through promoting PTEN-Akt signaling pathway in vitro after experimental traumatic brain injury. Brain Res. 2014;1582:12–20. 25108037 10.1016/j.brainres.2014.07.045

[43] Li D, Huang S, Yin Z, Zhu J, Ge X, Han Z, et al. Increases in miR-124-3p in Microglial Exosomes Confer Neuroprotective Effects by Targeting FIP200-Mediated Neuronal Autophagy Following Traumatic Brain Injury. Neurochem Res. 2019;448:1903–23. 10.1007/s11064-019-02825-1 31190315

[44] Yang L, Tao Y, Luo L, Zhang Y, Wang X, Meng X. Dengzhan Xixin injection derived from a traditional Chinese herb Erigeron breviscapus ameliorates cerebral ischemia/reperfusion injury in rats via modulation of mitophagy and mitochondrial apoptosis. J Ethnopharmacol. 2022;288:114988. 35032588 10.1016/j.jep.2022.114988

[45] Liu S, Lu C, Liu Y, Zhou X, Sun L, Gu Q, et al. Hyperbaric Oxygen Alleviates the Inflammatory Response Induced by LPS Through Inhibition of NF-κB/MAPKs-CCL2/CXCL1 Signaling Pathway in Cultured Astrocytes. Inflammation. 2018;416:2003–11. 10.1007/s10753-018-0843-2 30073566

[46] Karve IP, Taylor JM, Crack PJ. The contribution of astrocytes and microglia to traumatic brain injury. Br J Pharmacol. 2016;1734:692–702. 10.1111/bph.13125 PMC4742296 25752446

[47] Festa BP, Siddiqi FH, Jimenez-Sanchez M, Won H, Rob M, Djajadikerta A, et al. Microglial-to-neuronal CCR5 signaling regulates autophagy in neurodegeneration. Neuron. 2023;11113:2021–37.e12. 10.1016/j.neuron.2023.04.006 37105172

[48] Forslund MV, Roe C, Perrin PB, Sigurdardottir S, Lu J, Berntsen S, et al. The trajectories of overall disability in the first 5 years after moderate and severe traumatic brain injury. Brain Inj. 2017;313:329–35. 10.1080/02699052.2016.1255778 28095032

[49] Pu H, Ma C, Zhao Y, Wang Y, Zhang W, Miao W, et al. Intranasal delivery of interleukin-4 attenuates chronic cognitive deficits via beneficial microglial responses in experimental traumatic brain injury. J Cereb Blood Flow Metab. 2021;4111:2870–86. 10.1177/0271678X211028680 PMC8545055 34259069

[50] Lippert-Grüner M, Kuchta J, Hellmich M, Klug N. Neurobehavioural deficits after severe traumatic brain injury (TBI). Brain Inj. 2006;206:569–74. 10.1080/02699050600664467 16754282

[51] Xia Y, Pu H, Leak RK, Shi Y, Mu H, Hu X, et al. Tissue plasminogen activator promotes white matter integrity and functional recovery in a murine model of traumatic brain injury. Proc Natl Acad Sci USA. 2018;11539:E9230–e8. 10.1073/pnas.1810693115 PMC6166834 30201709

[52] Mukherjee S, Arisi GM, Mims K, Hollingsworth G, O’Neil K, Shapiro LA. Neuroinflammatory mechanisms of post-traumatic epilepsy. J Neuroinflammation. 2020;171:193. 10.1186/s12974-020-01854-w PMC7301453 32552898

[53] Jha RM, Kochanek PM, Simard JM. Pathophysiology and treatment of cerebral edema in traumatic brain injury. Neuropharmacology. 2019;145Pt B:230–46. 10.1016/j.neuropharm.2018.08.004 PMC6309515 30086289

[54] Keep RF, Andjelkovic AV, Xiang J, Stamatovic SM, Antonetti DA, Hua Y, et al. Brain endothelial cell junctions after cerebral hemorrhage: Changes, mechanisms and therapeutic targets. J Cereb Blood Flow Metab. 2018;388:1255–75. 10.1177/0271678X18774666 PMC6092767 29737222

[55] Allen CL, Bayraktutan U. Oxidative stress and its role in the pathogenesis of ischaemic stroke. Int J Stroke. 2009;46:461–70. 10.1111/j.1747-4949.2009.00387.x 19930058

[56] Brickler TR, Hazy A, Guilhaume Correa F, Dai R, Kowalski EJA, Dickerson R, et al. Angiopoietin/Tie2 Axis Regulates the Age-at-Injury Cerebrovascular Response to Traumatic Brain Injury. J Neurosci. 2018;3845:9618–34. 10.1523/JNEUROSCI.0914-18.2018 PMC6222064 30242049

[57] Hsiao CC, Vos E, van Gisbergen K, Hamann J. The adhesion G protein-coupled receptor GPR56/ADGRG1 in cytotoxic lymphocytes. Basic Clin Pharmacol Toxicol. 2023;1334:286–94. 10.1111/bcpt.13841 36750420

[58] Kumar A, Alvarez-Croda DM, Stoica BA, Faden AI, Loane DJ. Microglial/Macrophage Polarization Dynamics following Traumatic Brain Injury. J Neurotrauma. 2016;3319:1732–50. 10.1089/neu.2015.4268 PMC5065034 26486881

[59] Kumar A, Loane DJ. Neuroinflammation after traumatic brain injury: opportunities for therapeutic intervention. Brain Behav Immun. 2012;268:1191–201. 10.1016/j.bbi.2012.06.008 22728326

[60] Gyoneva S, Ransohoff RM. Inflammatory reaction after traumatic brain injury: therapeutic potential of targeting cell-cell communication by chemokines. Trends Pharmacol Sci. 2015;367:471–80. 10.1016/j.tips.2015.04.003 PMC4485943 25979813

[61] Henry RJ, Loane DJ. Targeting chronic and evolving neuroinflammation following traumatic brain injury to improve long-term outcomes: insights from microglial-depletion models. Neural Regen Res. 2021;165:976–7. 10.4103/1673-5374.297068 PMC8178770 33229740

[62] Donat CK, Scott G, Gentleman SM, Sastre M. Microglial Activation in Traumatic Brain Injury. Front Aging Neurosci. 2017;9:208. 28701948 10.3389/fnagi.2017.00208 PMC5487478

[63] Wang G, Zhang J, Hu X, Zhang L, Mao L, Jiang X, et al. Microglia/macrophage polarization dynamics in white matter after traumatic brain injury. J Cereb Blood Flow Metab. 2013;3312:1864–74. 10.1038/jcbfm.2013.146 PMC3851898 23942366

[64] Wang G, Shi Y, Jiang X, Leak RK, Hu X, Wu Y, et al. HDAC inhibition prevents white matter injury by modulating microglia/macrophage polarization through the GSK3β/PTEN/Akt axis. Proc Natl Acad Sci USA. 2015;1129:2853–8. 10.1073/pnas.1501441112 PMC4352818 25691750

[65] Ximerakis M, Lipnick SL, Innes BT, Simmons SK, Adiconis X, Dionne D, et al. Single-cell transcriptomic profiling of the aging mouse brain. Nat Neurosci. 2019;2210:1696–708. 10.1038/s41593-019-0491-3 31551601

[66] Plaza-Zabala A, Sierra-Torre V, Sierra A. Autophagy and microglia: novel partners in neurodegeneration and aging. Int J Mol Sci. 2017;18:598. 10.3390/ijms18030598 PMC5372614 28282924

[67] Park SJ, Frake RA, Karabiyik C, Son SM, Siddiqi FH, Bento CF, et al. Vinexin contributes to autophagic decline in brain ageing across species. Cell Death Differ. 2022;295:1055–70. 10.1038/s41418-021-00903-y PMC9090768 34848853

[68] Oppermann M. Chemokine receptor CCR5: insights into structure, function, and regulation. Cell Signal. 2004;1611:1201–10. 10.1016/j.cellsig.2004.04.007 15337520

[69] Boutet A, Salim H, Leclerc P, Tardieu M. Cellular expression of functional chemokine receptor CCR5 and CXCR4 in human embryonic neurons. Neurosci Lett. 2001;3112:105–8. 10.1016/s0304-3940(01)02149-8 11567789

[70] Westmoreland SV, Alvarez X, deBakker C, Aye P, Wilson ML, Williams KC, et al. Developmental expression patterns of CCR5 and CXCR4 in the rhesus macaque brain. J Neuroimmunol. 2002;1221-2:146–58. 10.1016/s0165-5728(01)00457-x 11777554

[71] Yan J, Xu W, Lenahan C, Huang L, Wen J, Li G, et al. CCR5 Activation Promotes NLRP1-Dependent Neuronal Pyroptosis via CCR5/PKA/CREB Pathway After Intracerebral Hemorrhage. Stroke. 2021;5212:4021–32. 10.1161/STROKEAHA.120.033285 PMC8607924 34719258

[72] Latinovic OS, Reitz M, Heredia A. CCR5 Inhibitors and HIV-1 Infection. J AIDS HIV Treat. 2019;11:1–5. 10.33696/AIDS.1.001 PMC6693856 31414081

[73] Tan Q, Zhu Y, Li J, Chen Z, Han GW, Kufareva I, et al. Structure of the CCR5 chemokine receptor-HIV entry inhibitor maraviroc complex. Science. 2013;3416152:1387–90. 10.1126/science.1241475 PMC3819204 24030490

[74] Vizurraga AL, Robertson MJ, Yu M, Skiniotis G, Tall GG. Hexahydroquinoline Derivatives Are Selective Agonists for the Adhesion G Protein-Coupled Receptor ADGRG1/GPR56. Mol Pharmacol. 2023;1041:28–41. 10.1124/molpharm.123.000688 PMC10289240 37290962

