# Supplementary material for: Genetic deletion of G protein-coupled receptor 56 aggravates traumatic brain injury through the microglial CCL3/4/5 upregulation targeted to CCR5
Source: Cell Death Dis. 2025 Mar 15;16(1):175. doi: 10.1038/s41419-025-07501-7 (PMC11910551; doi:10.1038/s41419-025-07501-7)

Full uncropped Gels and Blots images for Fig. 2F

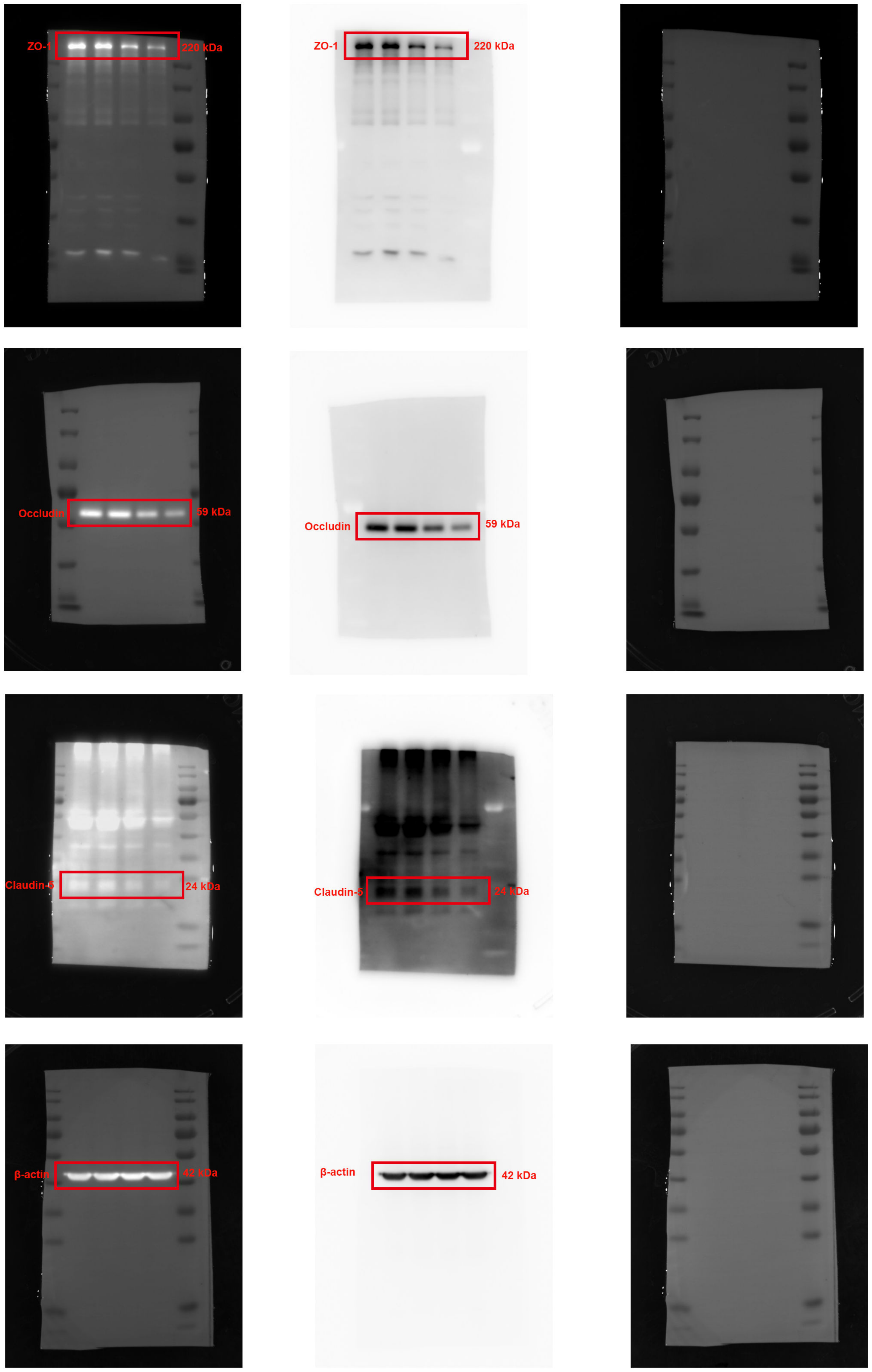

Full uncropped Gels and Blots images for Fig. 6A

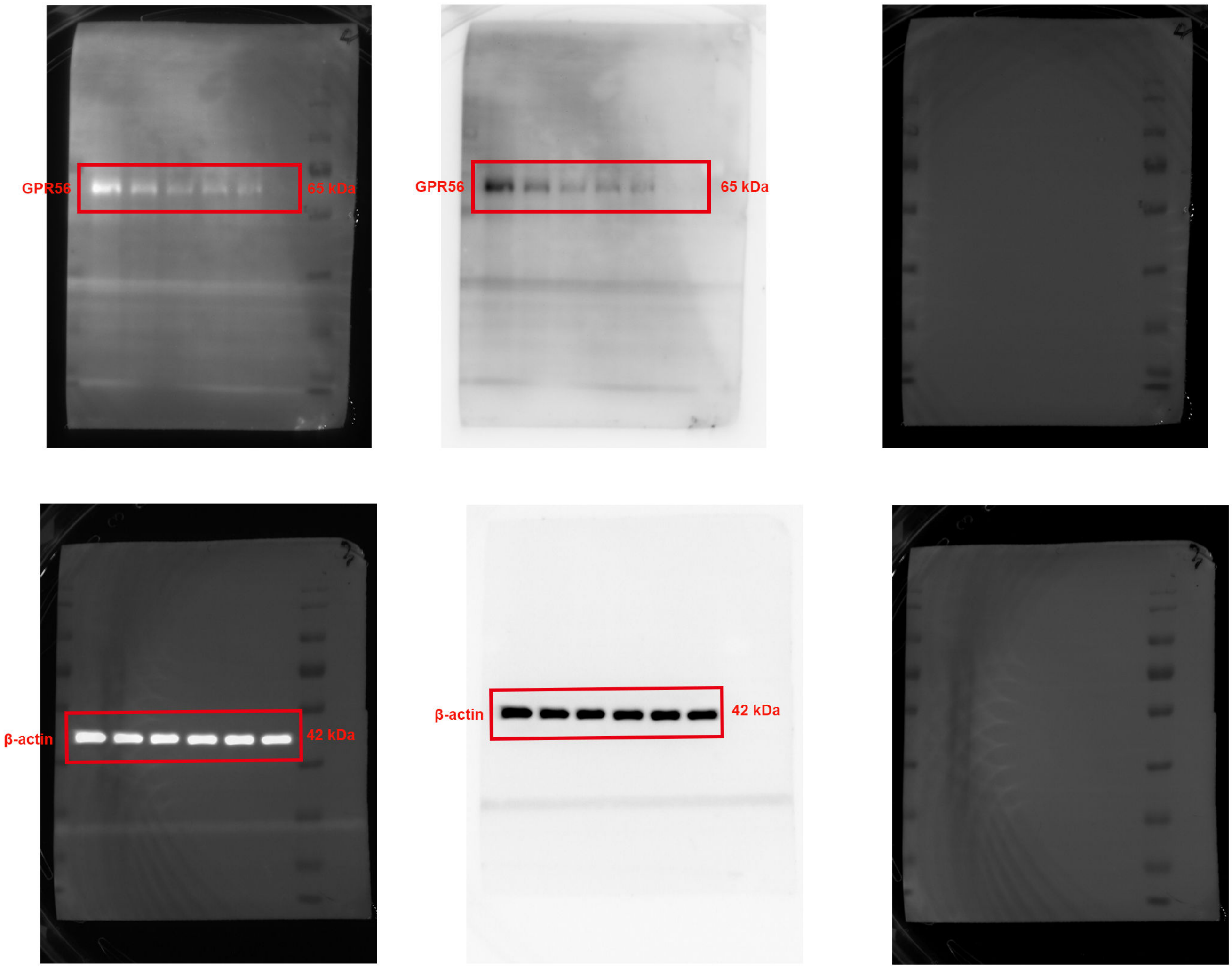

Full uncropped Gels and Blots images for Fig. 7A

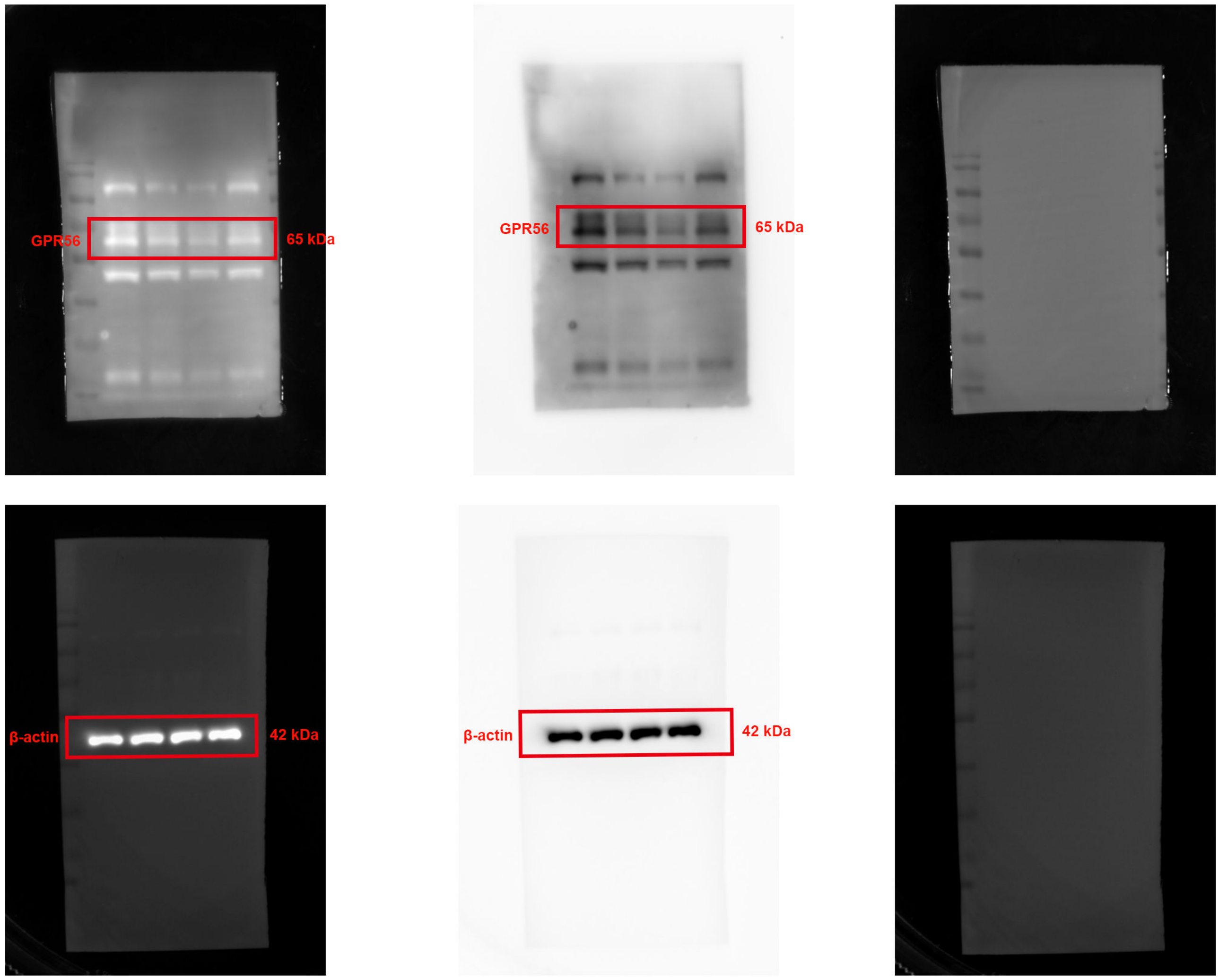

Full uncropped Gels and Blots images for Fig. 8C

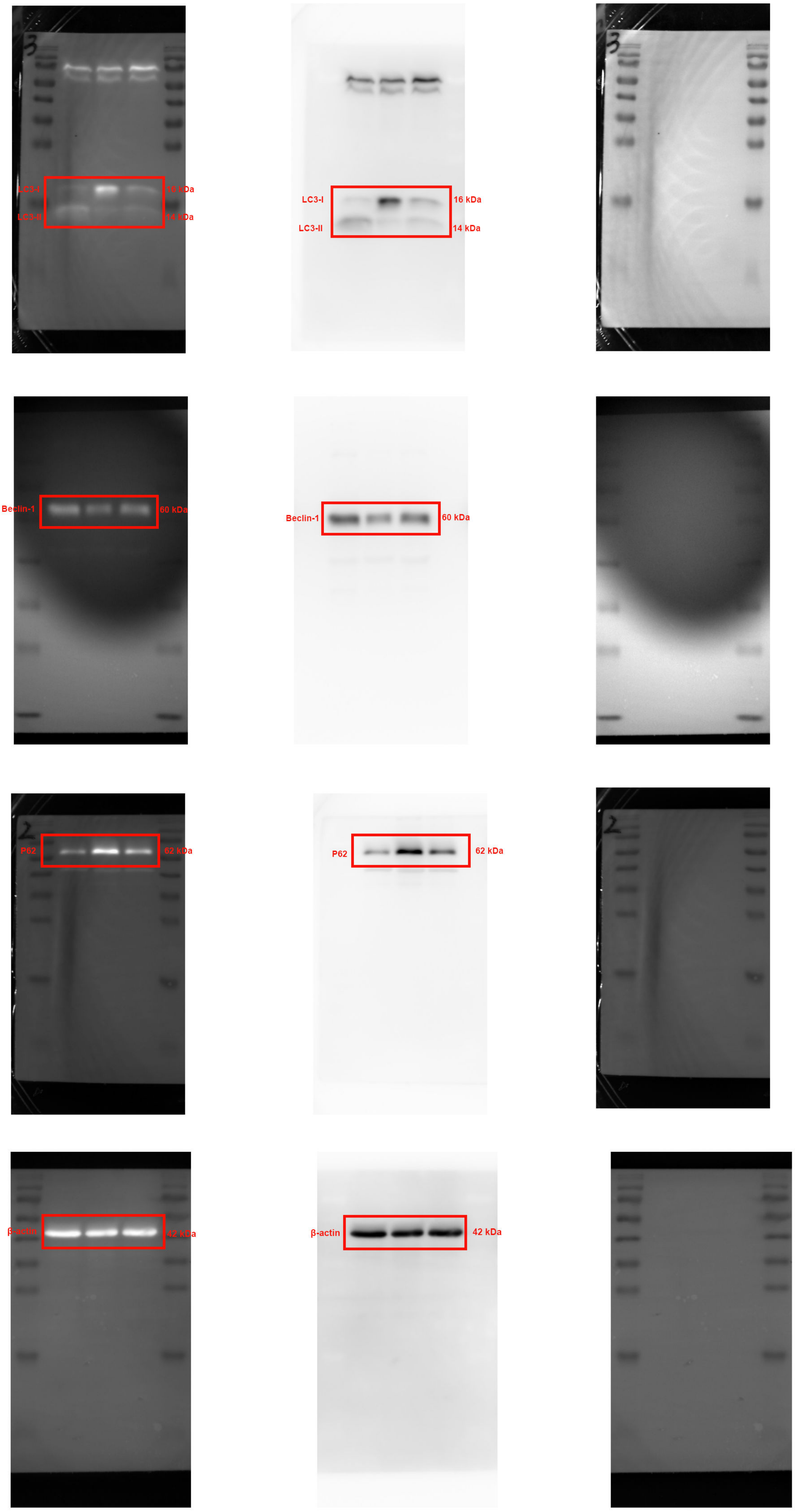

Full uncropped Gels and Blots images for Fig. 8I

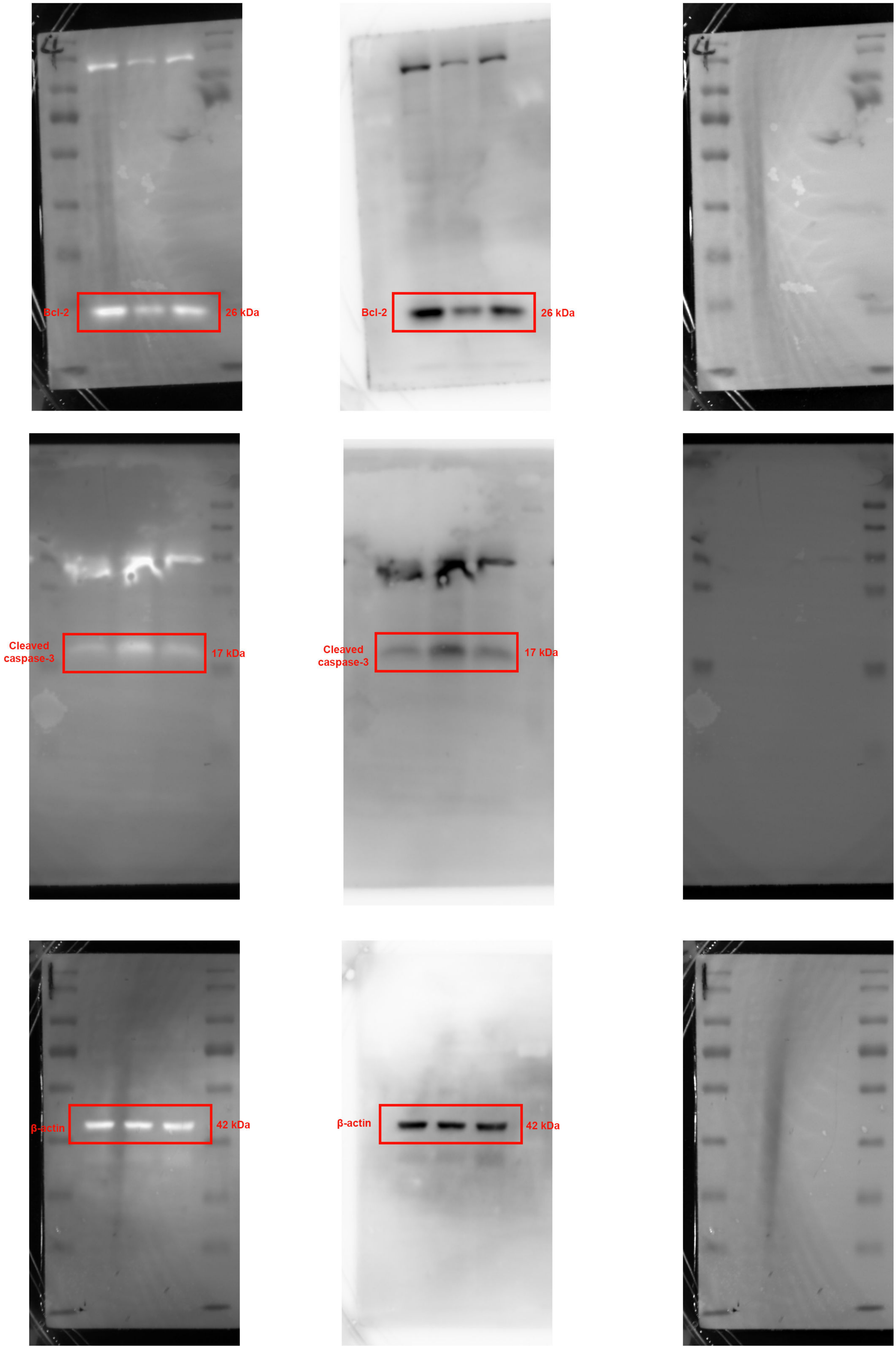

Supplement: Supplementary file 1 — Full uncropped Gels and Blots images [file 41419_2025_7501_MOESM1_ESM.pdf]
