# Supplementary material for: Genetic deletion of G protein-coupled receptor 56 aggravates traumatic brain injury through the microglial CCL3/4/5 upregulation targeted to CCR5
Source: Cell Death Dis. 2025 Mar 15;16(1):175. doi: 10.1038/s41419-025-07501-7 (PMC11910551; doi:10.1038/s41419-025-07501-7)
Supplement: Supplementary file 2 — Supplemental figures [file 41419_2025_7501_MOESM2_ESM.docx]

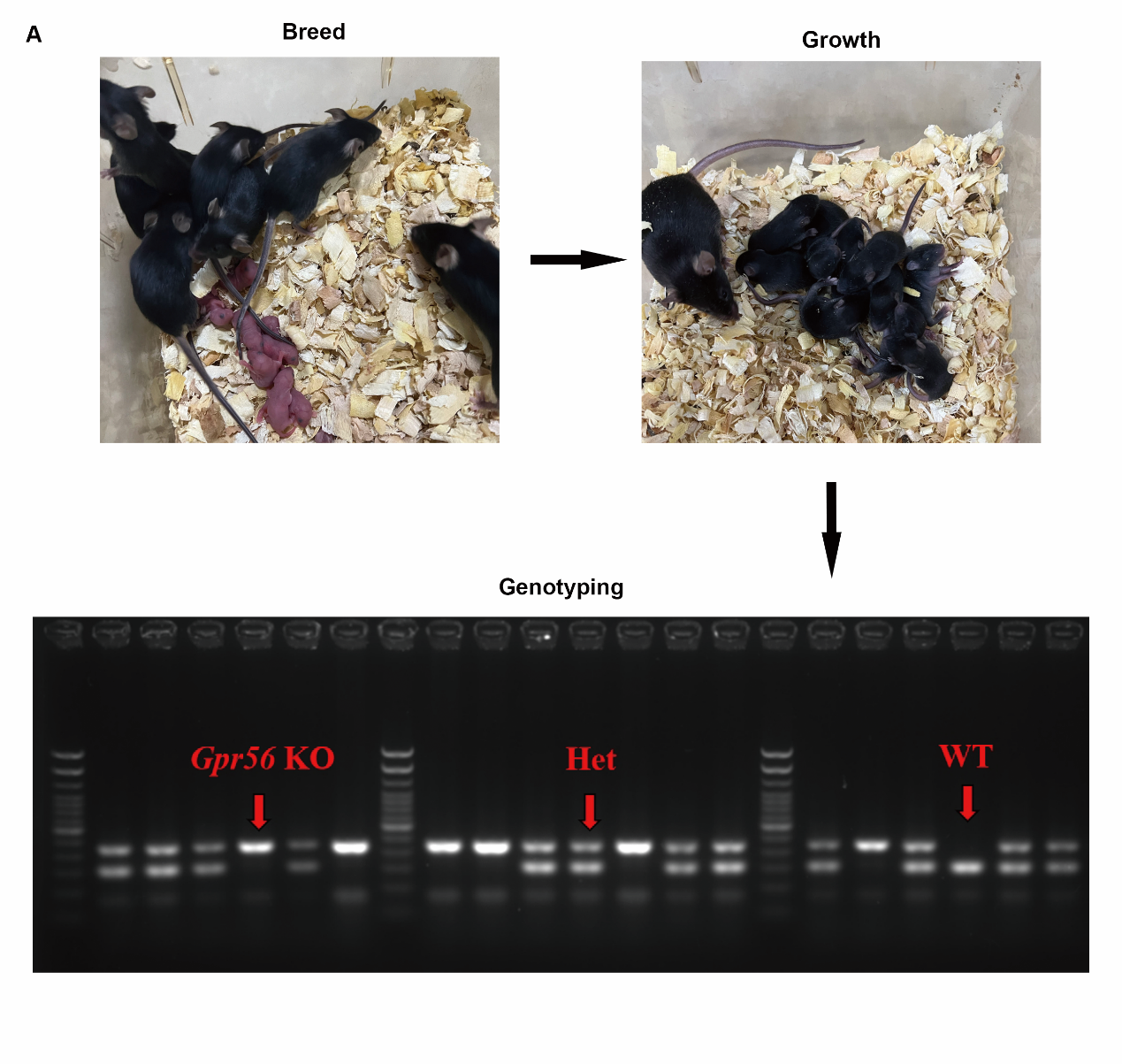


**Fig. S1. Reproductive breeding and genotype identification of mice. (A)** *Gpr56* KO mice were obtained through mating and reproduction of het mice, and genotyped through PCR.


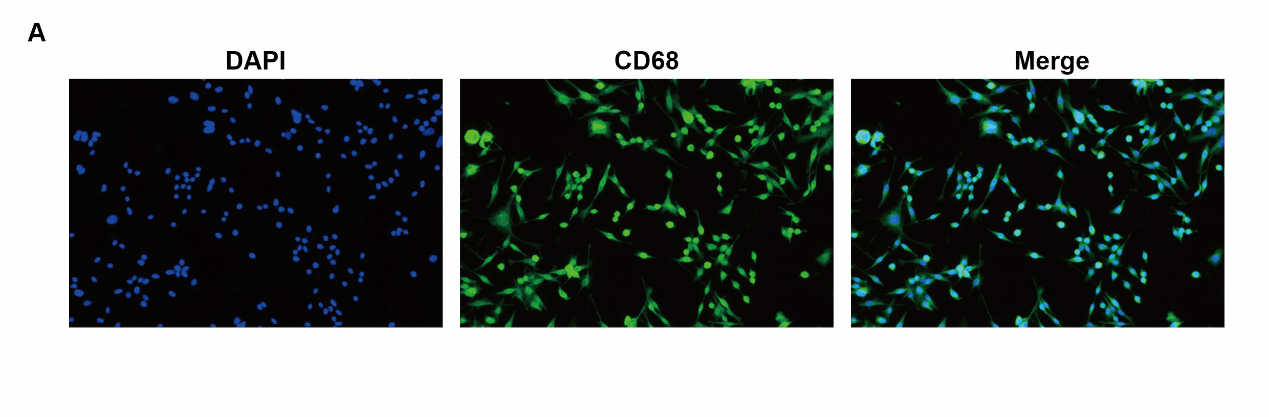


**Fig. S2. Identification of primary microglia. (A)** The primary microglia were identified through immunofluorescence by CD68.


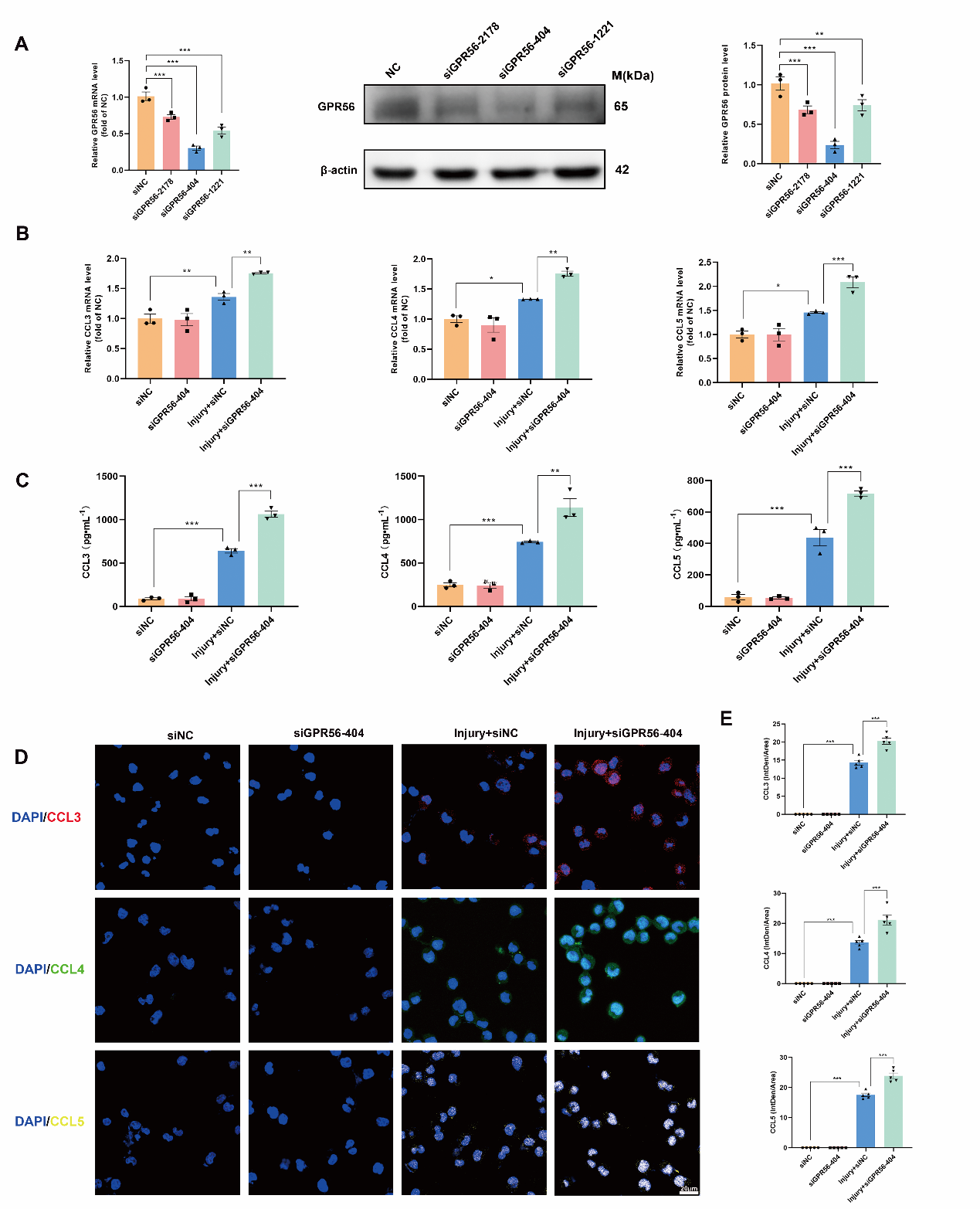


**Fig. S3. Downregulation of GPR56 in cultured microglia promotes chemokines secretion. (A)** The qRT-PCR and western blot analysis revealed the efficiency of GPR56 downregulation in Bv2 microglial cells by different GPR56 siRNAs in mRNA and protein levels. **(B)** Changes in the Bv2 microglial cells mRNA expression levels of pro-inflammatory chemokines CCL3, CCL4, and CCL5 in experimental groups. **(C)** ELISA results of chemokines CCL3, CCL4, and CCL5 in experimental groups. **(D)** Representative confocal fluorescence images of chemokines CCL3, CCL4, and CCL5 in Bv2 microglial cells in experimental groups. **(E)** Quantitative analysis of CCL3/4/5 fluorescence intensities. Scale bars: 20 µm. The *in vitro* experiments were repeated three times. **p* < 0.05, ***p* < 0.01, ****p* < 0.001.
